# Exploring the transcriptome of hormone-naive multifocal prostate cancer and matched lymph node metastases

**DOI:** 10.1038/s41416-018-0321-5

**Published:** 2018-11-19

**Authors:** Linnéa Schmidt, Mia Møller, Christa Haldrup, Siri H. Strand, Søren Vang, Jakob Hedegaard, Søren Høyer, Michael Borre, Torben Ørntoft, Karina Dalsgaard Sørensen

**Affiliations:** 1 Department of Molecular Medicine, Aarhus University Hospital, Aarhus, Denmark; 2 Department of Pathology, Aarhus University Hospital, Aarhus, Denmark; 3 Department of Urology, Aarhus University Hospital, Aarhus, Denmark

**Keywords:** Prognostic markers, Cancer genomics, Prostate cancer

## Abstract

**Background:**

The current inability to predict whether a primary prostate cancer (PC) will progress to metastatic disease leads to overtreatment of indolent PCs as well as undertreatment of aggressive PCs. Here, we explored the transcriptional changes associated with metastatic progression of multifocal hormone-naive PC.

**Methods:**

Using total RNA-sequencing, we analysed laser micro-dissected primary PC foci (*n* = 23), adjacent normal prostate tissue samples (*n* = 23) and lymph node metastases (*n* = 9) from ten hormone-naive PC patients. Genes important for PC progression were identified using differential gene expression and clustering analysis. From these, two multi-gene-based expression signatures (models) were developed, and their prognostic potential was evaluated using Cox-regression and Kaplan–Meier analyses in three independent radical prostatectomy (RP) cohorts (>650 patients).

**Results:**

We identified several novel PC-associated transcripts deregulated during PC progression, and these transcripts were used to develop two novel gene-expression-based prognostic models. The models showed independent prognostic potential in three RP cohorts (*n* = 405, *n* = 107 and *n* = 91), using biochemical recurrence after RP as the primary clinical endpoint.

**Conclusions:**

We identified several transcripts deregulated during PC progression and developed two new prognostic models for PC risk stratification, each of which showed independent prognostic value beyond routine clinicopathological factors in three independent RP cohorts.

## Introduction

Localised prostate cancer (PC) is generally curable by radical prostatectomy or radiation therapy, whereas disseminated PC cannot be cured.^1^ Frequent side effects of primary PC treatment include incontinence and impotence, and it is therefore highly important to distinguish localised PCs that will develop into aggressive disease from those that will not, in order to guide treatment decisions. The prognostic tools currently used to assess PC aggressiveness (primarily Gleason score, TNM stage, and pre-operative serum PSA levels) are suboptimal, leading to overtreatment of indolent tumours and insufficient or delayed treatment of aggressive tumours.^1^ Existing knowledge of the molecular alterations that drive progression towards aggressive metastatic PC is insufficient. In addition, the multifocality and molecular heterogeneity often seen in PC, complicates biomarker development.^2,3^ Thus, new and more accurate biomarkers are urgently needed to solve these clinical challenges.

Primary PC often presents as a multifocal disease, harboring several morphologically and often also clonally distinct tumour foci.^4–6^ Despite the multifocal heterogeneity of the primary foci, studies have shown that many distant metastases in the same patient share a majority of genetic alterations, suggesting that the metastatic cells originate from one primary monoclonal focus.^5,6^ However, evidence of polyclonal seeding has also been reported, suggesting that both mono- and polyclonal seeding may occur during the progression of PC.^7^

Previous research on metastatic PC or castration-resistant PC (CRPC) has generally been based on metastatic samples from patients pre-treated with androgen deprivation therapy (ADT) and/or additional secondary endocrine or chemotherapy, i.e. heavily pre-treated patients.^5–18^ While these earlier studies provided new knowledge on how treatment affects tumour progression, they did not directly assess the initiating events associated with metastatic progression in primary hormone-naive PC. To our knowledge, only one study including metastatic tissue from hormone-naive PC patients has been published, in which analysis of the DNA methylome of multiple PC samples and metastatic tissue led the authors to propose that heterogeneity in methylation patterns mirrors the genomic/clonal evolution of PC.^19^ To our knowledge, studies on the transcriptional level have not yet been performed, which could give a deeper insight into the functional genome, by directly investigating gene-expression activity.

In addition to the previous development of gene-expression signatures for PC and PC aggressiveness,^20–29^ comparison of gene-expression patterns in different tissue types during PC progression (benign tissue, cancerous tissue and metastatic tissue) should enable the discovery of gene signature profiles that can be associated with functionally important steps in the progression of aggressive PC. In this study, we sought to provide new knowledge on differentially expressed genes during the progression of aggressive PC. We analysed the transcriptome of multiple paired tissue samples from lymph node metastases, primary tumours, and adjacent normal samples from ten hormone-naive PC patients using total RNA-sequencing (RNA-Seq). Moreover, we used these data to develop two expression-based multi-gene models, which displayed prognostic potential in three radical prostatectomy (RP) cohorts independent of clinicopathological variables.

## Materials and methods

### Patient material

#### Discovery cohort: Hormone-naive prostate cancer patients with matched primary tumour and lymph node metastasis samples

**Fig. 1:**
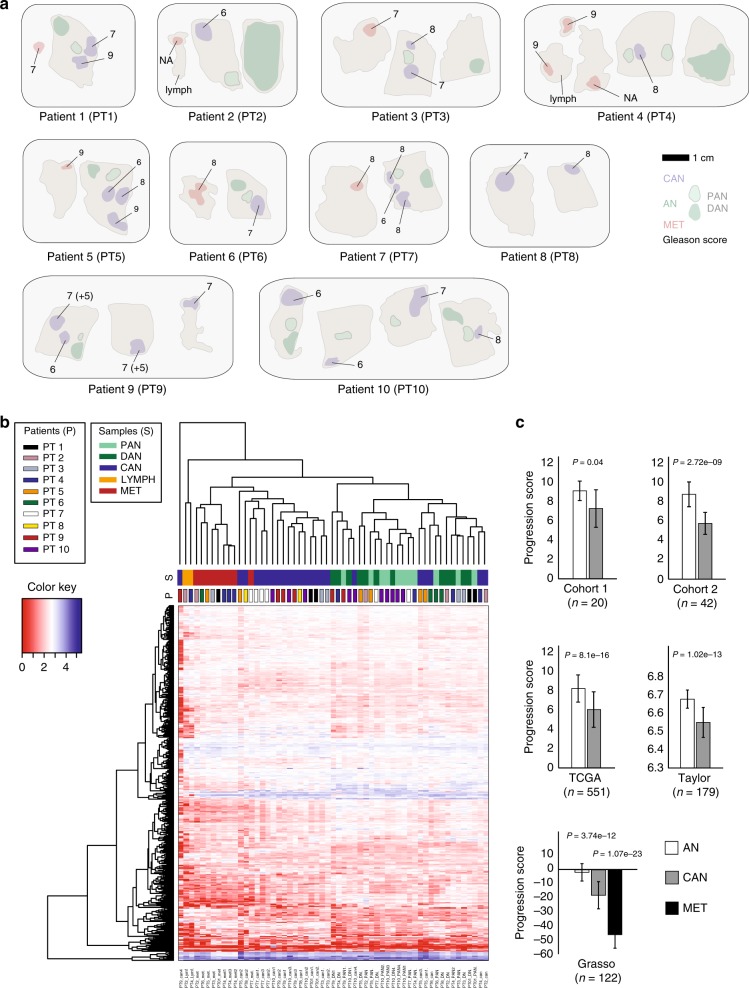
Workflow describing sample preparation. **a** An illustration of HE stained sections of prostate and lymph node FFPE tissue from ten radical prostatectomy patients (PT1-PT10). Firstly, FFPE tissue blocks were sectioned for HE staining and for laser microdissection. Secondly, marked tissue areas (colored regions in figure) were used for laser microdissection, RNA extraction, and RNA-sequencing. DAN distant adjacent normal, PAN proximal adjacent normal, CAN primary tumor, MET lymph node metastasis, LYMPH non-malignant lymph node. NA not available. **b** Heatmap based on RNA expression for all samples. Hierarchical clustering of patient samples, using the 500 most variable transcripts, revealed a distinct clustering pattern where samples types clustered closer together than intrapatient samples. Rows correspond to patient samples (patient numbers and sample types; CAN, DAN, PAN, MET and LYMPH are illustrated by the colored bars above the heatmap). Columns correspond to the 500 transcripts with the largest variation between samples. **c** Progression scores in five external PC patient cohorts (Methods). The sum of the expression values of the genes included were validated to be lower in the more aggressive tissue types in Strand (6 AN and 14 CAN), Haldrup (13 AN and 29 CAN), TCGA (52 AN and 499 CAN), Taylor (29 AN and 150 CAN) and in Grasso (28 AN, 59 CAN, and 35 MET), as assessed by two-sided *t*-tests (overlap of the 19 genes in each cohort can be found in Methods)

We obtained formalin-fixed paraffin-embedded (FFPE) whole-prostate and lymph node tissue blocks from ten hormone-naive PC patients who underwent radical prostatectomy (RP) for histologically verified primary PC at the Department of Urology, Aarhus University Hospital, Denmark (2002-2009) (see Table 1 for clinicopathological information). An expert pathologist evaluated three hematoxylin and eosin-stained sections (top, middle, and bottom) from all tissue blocks, and marked areas of distant adjacent normal (DAN; i.e. benign prostate epithelial tissue > 3 mm away from the nearest PC focus), proximal adjacent normal (PAN, i.e. benign epithelial tissue < 1 mm away from a PC focus), primary PC tissue (CAN), and lymph node metastasis tissue (MET) samples, as previously described^30^ (Figure S1A-B). In addition, non-malignant lymph node tissue samples (LYMPH) were included for two patients (PT2 and PT4). For this study, a total of 31 FFPE tissue blocks (64 areas) were used for Laser Microdissection (LMD), and RNA extraction was performed for each of these 64 areas (Fig. 1a). Seven samples with low quality sequencing libraries were discarded, leaving 57 samples eligible for downstream analysis (10 × DAN, 13 × PAN, 23 × CAN, 9 × MET, 2 × LYMPH; Table 1). Experimental procedures for total RNA extraction and RNA-Seq are described in Supplementary methods. RNA-Seq summary statistics are given in Table S1.

**Table 1:** Clinicopathological characteristics for patients 1-10 (PT1-PT10) and final RNA-seq samples

Patient	Sample	PT1	PT2	PT3	PT4	PT5	PT6	PT7	PT8	PT9	PT10
DAN		1	1	1	1	1	1	1	0	1	2
PAN		1	1	1	2	1	1	1	0	1	4
CAN		2	1	2	1	3	1	3	2	4	4
MET		1	1	1	3	1	1	1	0	0	0
LYMPH		0	1	0	1	0	0	0	0	0	0
Age at RP, years		61	51	67	49	63	64	69	65	52	68
Pre-operative PSA, ng/mL		21.0	28.9	18.1	47.1	5.6	22.6	155	7.0	20.6	7.2
Pathological Gleason Score		7	6	7	8	7	7	7	7	7	7
	CAN 1	4 + 3,	3 + 3	4 + 4,	4 + 3	3 + 3,	3 + 4	4 + 4,	3 + 4	3 + 4 + 5,	3 + 3,
	CAN 2	4 + 5		4 + 3		4 + 4,		3 + 3,	4 + 4	3 + 3,	3 + 3,
	CAN 3					4 + 5		4 + 4		3 + 4 + 5,	3 + 4,
Gleason grades^a^										3 + 4	4 + 4
	MET 1	4 + 3		4 + 3	4 + 5	4 + 5	4 + 4	4 + 4			
	MET 2				4 + 5						
	MET 3				—						
pT		pT3	pT3	pT3	pT3	pT3	pT3	pT3	pT3	pT3	pT3
pN		pN1	pN1	pN1	pN1	pN1	pN1	pN1	pN1	pN1	pN1

*DAN* Distant adjacent normal, *PAN* proximal adjacent normal, *CAN* primary tumour, *MET* lymph node metastasis, *LYMPH* non-malignant lymph node, *pT* pathological tumour stage, *pN* pathological lymph node status

^a^Gleason grades were determined for each included focus from the primary tumour and MET samples

#### Prostate cancer sample sets with existing transcriptomic profiling data used for external validation

To be able to validate candidate transcripts and multi-transcript models identified in the discovery cohort, we collected gene-expression profiling data and clinical data from six publicly available PC cohorts of different sizes, characteristics and geographical origins. From two RP patient sets, we had RNA-Seq data (Fragments Per Kilobase of transcript per Million mapped reads, FPKM) for 6 AN and 14 CAN samples (Strand cohort), respectively 13 AN and 29 CAN samples (Haldrup cohort).^31,32^ The Grasso cohort^14^ included 28 AN, 59 CAN and 35 metastatic castrate-resistant prostate cancer tissue samples from 122 PC patients, profiled on the Whole Human Genome Microarray 4 × 44 K and Whole Human Genome Oligo Microarray (downloaded from the Gene Expression Omnibus (GEO) database, accession number GSE35988). RNA-Seq data (FPKM) from the TCGA consortium including 551 PC patients (52 AN and 499 CAN samples) was downloaded from the GDC data portal, NIH National Cancer Institute, USA.^33^ The Taylor cohort^34^ included 29 AN and 150 CAN samples from 179 PC patients, profiled on the Affymetrix Human Exon 1.0 ST Array (downloaded from GEO, accession number GSE21036).

For testing the prognostic multi-gene-expression models (described below), we used primary PC tissue samples (CAN) from RP patients in the TCGA (*n* = 499), Taylor (*n* = 150) and Long (*n* = 106)^24^ cohorts. In the TCGA cohort, FPKM values for the overlapping genes of interest, i.e. genes included in the two prognostic models, and clinical information  were available for 477 patients, which were included in the final analyses. In the Taylor cohort, all patients receiving post-endocrine treatment (*n* = 43) were excluded from the analysis (this information was unavailable in the TCGA and Long cohorts), leaving 107 patients eligible for final analyses. The Long cohort was profiled using RNA-Seq and downloaded from the GEO database (accession number GSE54460). After removing duplicates (*n* = 6) and patients with 0 days to post-operative BCR (*n* = 9), a total of 91 patients in the Long cohort were eligible for further analysis. Clinicopathological information on TCGA, Taylor, and Long can be found in Table S2.

### Prognostic gene models

Differential gene-expression analysis was performed in our discovery cohort and enabled us (i) to investigate genes associated with the progression from localised PC to metastatic disease, by exploring the expression levels in AN, CAN and MET samples, and; (ii) to define one of several PC foci in the same patient, a likely “seeding focus”, which was proposed to be responsible for seeding the metastasis. Two different strategies were used to develop the progression model and the seeding model.

#### Progression model

To identify transcripts associated with the progression of primary PC to metastasis, differential gene-expression analysis was performed for the AN group versus CAN group, and for the CAN group versus MET group. Transcripts with an expression pattern in the same direction of log fold change below 2 or above 2 from AN to CAN, and furthermore from CAN to MET (FDR < 0.05) were considered for further analysis. We found 19 downregulated transcripts and no upregulated transcripts using this strategy (Table S3). For testing in five of the six validation cohorts (above) the 19 genes were summarised in each cohort, and log2-transformed to give a progression score for each patient. Due to different platforms used (Microarray or RNA-Seq), out of the 19 genes included in the progression model, transcriptional expression data were available for 19 genes (100%) in the Strand cohort, for 19 genes (100%) in the Haldrup cohort, for 17 genes (89.5%) in TCGA, for 12 genes (70.6%) in the Taylor cohort, for 15 genes (78.9%) in the Long cohort, and for 14 genes (73.7%) in the Grasso cohort. For validation of the prognostic potential, a weighted model was developed from the 19 genes (below), which was tested in the TCGA, Taylor and Long cohorts (below) using Cox-regression and Kaplan–Meier analyses.

#### Seeding model

Four patients with multifocal PC (PT1, PT3, PT5 and PT7) had two or more primary PC samples and a matched metastatic sample analysed by RNA-Seq. Thus, these patients were eligible for analysis to find the PC focus most likely to be the origin of the metastasis in each patient. Hence, as a first step to identify genes for the seeding model, the 500, 100 and 50 most variable transcripts were clustered (heatmap.2 function in R^35^) to identify the CAN sample which clustered with the MET sample. A likely “seeding focus” could be identified in two patients (PT5 and PT7). These “seeding focus” samples were compared, using gene-expression analysis in the edgeR package,^35^ to the four likely “non-seeding focus” samples from the same patients. Genes with significant differential expression (FDR < 0.01) were considered to be important for the seeding process (*n* = 20, Table S4). For validation of the prognostic potential of the seeding model, the TCGA, Taylor and Long cohorts were used.^24,33,34^ Due to different platforms used (Microarray or RNA-Seq), out of the 20 genes included in the seeding model, transcriptional expression data were available for 20 genes (100%) in the TCGA cohort, for 13 genes (60%) in the Taylor cohort and for 18 genes (90%) in the Long cohort.

#### Weighted models

To be able to test the prognostic potential of the genes found to be associated with the progression of PC (*n* = 19) and the genes found to likely be involved in seeding of the metastasis (*n* = 20), two prognostic models were calculated from the normalised gene-expression values from the 19 genes (progression model), and the 20 genes (seeding model), respectively. The models were calculated in each of the three cohorts (TCGA, Taylor, and Long) where an analysis of prognostic potential was possible, i.e. cohorts with follow-up data including BCR and time to BCR. Gene-expression values were multiplied by the regression coefficient from each gene in multivariate Cox-regression analysis, and the following weighted expression values were summarised into a model score for each patient. The final model score for each patient was determined by dividing the individual score with the standard deviation of the scores for all patients.

### Gene set enrichment analysis (GSEA)

Differentially expressed transcripts and genes from the progression model were investigated for gene set enrichments using the GSEA software 3.0, and the online Molecular Signatures Database (MSigDB) (http://software.broadinstitute.org/gsea),^36,37^ respectively. Gene lists with FDR < 0.25 were considered statistically significant. FPKM values used for the analysis were filtered to include only transcripts expressed at >1 FPKM in at least 50% of each subgroup (CAN, AN, MET, LYMPH), resulting in 11,017 transcripts available for GSEA. The same 11,017 transcripts were used when comparing PAN and DAN samples using GSEA.

### Statistical analyses

All statistical analyses were conducted in R^35^ unless stated otherwise. False discovery rate (FDR) and *P* values < 0.05 were considered significant, unless stated otherwise. Associations between the seeding model and the progression model and clinicopathological parameters were assessed using the Wilcoxon rank-sum test. For evaluation of prognostic potential, the primary clinical endpoint was BCR-free survival (RFS) after RP. Patients not having experienced BCR were censored at their last PSA test. For RFS analyses, we performed uni- and multivariate Cox-regression analyses as well as Kaplan–Meier analyses and two-sided log-rank tests using the survival package in R.^35^ For Kaplan–Meier analysis, the cutoff (fraction) defined in TCGA was applied to the Taylor and Long cohorts for the progression and seeding model, respectively. For multivariate analysis, only clinicopathological parameters significant in univariate analysis were included. Variables failing in this analysis were excluded from the final multivariate model through stepwise backward selection. Predictive accuracy was determined using Harrell’s concordance index (C-index).

## Results

### RNA-Seq of 57 matched samples from 10 hormone-naive prostate cancer patients

In order to investigate the gene-expression changes in metastatic PC tissue samples compared to primary tumour samples and adjacent normal prostate tissue samples, we performed LMD and RNA-Seq on multiple matched tissue samples from each of ten hormone-naive PC patients with metastatic disease (PT1-PT10, Fig. 1a and Figure S1). Matched samples of distant adjacent normal tissue (DAN), proximal adjacent normal (PAN), primary tumour tissue (CAN), and lymph node metastases (MET) were included for each patient (Fig. 1a). For two patients, non-malignant lymph node tissue samples (LYMPH) were also included (Fig. 1a). After QC, a total of 57 tissue samples were successfully sequenced (10 DAN, 13 PAN, 23 CAN, 9 MET, 2 LYMPH). Four patients had more than one primary PC focus included in the final data analysis. Gleason scores were determined for each metastatic sample and for each primary PC focus, and were highly heterogeneous within patients, e.g. Gleason scores 6, 8 and 9 were found in three distinct PC foci from PT5 (Table 1).

### Clustering revealed interpatient sample types more similar than intrapatient samples

To investigate the similarity of our samples, we performed clustering analysis of gene expression from all samples. We observed that sample types clustered together (CAN, MET, and an adjacent normal cluster of DAN and PAN samples), and the two non-malignant LYMPH samples clustered together. Thus, interpatient sample types were more similar than intrapatient samples (Fig. 1b). The clustering of samples by tissue types rather than by individual patient is in contrast to the patient wise clustering reported in a methylome study of multiple paired samples from hormone-naive PC patients.^19^

### Differential expression analysis

To identify genes differentially expressed between sample types (DAN, PAN, CAN, MET, LYMPH), we performed group-wise comparisons of gene expression. We initially looked for differential gene expression between the DAN and PAN sample group and found only 7 significantly differentially expressed transcripts (FDR < 0.05) out of more than 23,000 transcripts investigated (Table S5). Still, GSEA revealed a negative enrichment score for HALLMARK_P53_PATHWAY and HALLMARK_NOTCH_PATHWAY in PAN samples compared to DAN samples (FDR < 0.25, Table S6). Nevertheless, based on their highly similar expression profiles, we collapsed the DAN and PAN sample groups to an adjacent normal (AN) sample group for all subsequent analyses.

Next, we proceeded to evaluate differentially expressed genes between the AN and CAN samples, as well as between the CAN and MET samples. Among the top 20 most significantly upregulated transcripts in CAN versus AN samples, 45% were genes which have previously been studied in relation to PC biology and/or progression of PC (PC-associated genes) (Table S7), e.g. *ERG* and *AMACR*. The top 20 most significantly differentially downregulated transcripts in CAN versus AN samples, comprised 65% PC-associated genes, e.g. the keratin *KRT5* and Tumour Protein *TP63*. To identify classes of genes that were over or under-represented between sample types, GSEA was performed between the CAN and AN. The differential gene-expression results were corroborated by GSEA, e.g. a significant enrichment of HALLMARK_ANDROGEN_RESPONSE (FDR < 0.01, NES = 2.41; Table S8). Accordingly, a significant enrichment of the prostate-specific gene set “TOMLINS_PROSTATE_CANCER_UP” gene set was observed in CAN samples versus AN samples (Figure S2A).^38^ When exploring differential expression profiles for upregulated transcripts in CAN versus MET samples, only 10% of identified genes had previously been studied in PC (PC-associated genes) (Table S9). In contrast, for the downregulated transcripts, 60% of the transcripts were previously reported as PC-associated transcripts, exemplified by CNN1 (CYR61), SPOC3 and ACTG2 (Table S9). Based on these observations, a downregulation of transcripts during both the cancerous (AN to CAN) and metastatic (CAN to MET) processes were more pronounced compared to an upregulation of transcripts, suggesting that a loss of transcriptional activity is more common than upregulation of specific genes in the progression of PC.

### Genes involved in prostate cancer progression

We moved on to look for up- or downregulated transcripts associated with the malignant progression from AN to CAN, and further from CAN to MET. Using stringent criteria (Methods), we found no significantly upregulated transcripts, but 19 significantly downregulated transcripts from AN to CAN, and further downregulated from CAN to MET (Table S3). Of these 19 transcripts, five were non-coding RNAs and 14 were protein coding. For functional evaluation, we used GSEA to search for overrepresentation of the 19 genes in gene lists published by others (Methods). Interestingly, three of the top four identified gene lists included overlapping genes (*KRT15, KRT5, KRT23, COL13A1, FLRT3, MUC4, SMOC1, KCTD14* and *RBFOX3*) downregulated during PC development and progression (Table S10).

Further, the 19 genes were summarised to a progression score and analysed in five additional cohorts including Strand (*n* = 20), Haldrup (*n* = 42), Grasso (*n* = 122), TCGA (*n* = 551) and Taylor (*n* = 179). Four of the five cohorts included AN and CAN samples, while the Grasso cohort also included metastatic PC samples. Since the genes were found to be downregulated during cancerous and metastatic progression of PC, we hypothesised that the score should reflect this trend also in these external cohorts. Indeed, the progression score was significantly lower in CAN samples compared to AN samples in all five cohorts, as well as significantly lower in metastatic samples compared to cancer samples in the Grasso cohort (Fig. 1c). Thus, these results indicate that the identified genes are associated with PC progression in several external independent cohorts of different sizes and origins.

**Fig. 2:**
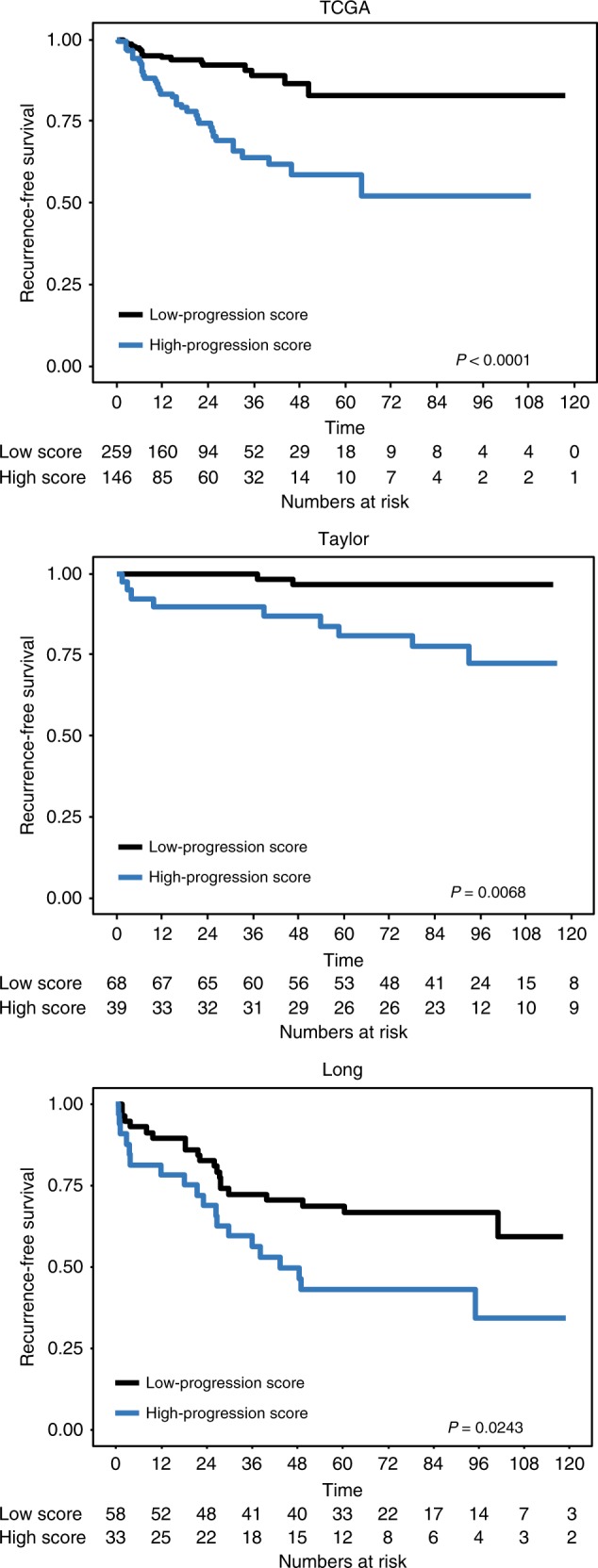
Kaplan–Meier analysis of the progression model in the TCGA, Taylor and Long cohorts. Kaplan–Meier analysis of the progression model in TCGA (top), Taylor (middle), and Long (bottom) cohorts

To test the prognostic potential of the 19 genes, three cohorts with clinical follow-up data were used (TCGA, Taylor, and Long). We used the gene-expression values to calculate a weighted progression model from available genes in each of the RP cohorts, with BCR as the clinical endpoint. When tested in Cox-regression analysis, the progression model was a significant predictor of BCR using univariate analysis in all three cohorts (TCGA: Hazard Ratio (HR) 2.61, *P* < 0.001; Taylor: HR 5.66, *P* = 0.0008; and Long: HR 2.33, *P* = 0.0068), and in multivariate Cox-regression analysis in TCGA and Taylor et al. (TCGA: HR 2.09, *P* *=* 0.0026; Taylor: HR 5.64, *P* *=* 0.0006) (Table 2). In TCGA, the C-index increased from 0.673 to 0.725, and in Taylor from 0.653 to 0.87, when adding the progression model to the multivariate model. Using Kaplan–Meier analysis, the progression model could successfully divide patients in high and low risk to experience BCR in all three cohorts (TCGA, *P* < 0.0001; Taylor, *P* = 0.0068; Long, *P* = 0.0243, log-rank test, Fig. 2). The cutoff for Kaplan–Meier analysis for Taylor et al., and for Long et al. was set using the fractions defined in the TCGA cohort. Furthermore, when assessing the importance of the progression model on clinicopathological variables in these three cohorts, a high progression model score was significantly associated with higher Gleason score (>7 vs. ≤7), advanced tumour stage (pT3 vs. pT2), higher pre-operative PSA values (PSA ≥ 10 ng/mL vs. <10 ng/mL), and positive nodal status at surgery in TCGA, and with higher Gleason score (>7 vs. ≤7) in Taylor (Wilcoxon rank-sum test; Figure S3A). No association between the progression model and clinicopathological variables in Long were observed (Wilcoxon rank-sum test; Figure S3A).

**Table 2:** Progression and seeding models in uni- and multivariate Cox-regression analysis in three cohorts (TCGA,Taylor, and Long)

Progression model								
		Univariate			Multivariate			
Variable	Characteristics	HR	*P*-value	*C-*index	HR	*P-*value	C-index	C-index*
TCGA (*n* = 405, 58 BCR)								
PSA	Continuous	1.03 (1.01–1.04)	**4.60E−03**	0.610	—	—	—	—
Margin status	Pos. vs. neg.	1.30 (0.77–2.19)	3.30E−01	0.519	—	—	—	—
Gleason score	>7 vs. ≤7	3.79 (2.10–6.84)	**9.38E−** **06**	0.642	2.07 (1.10–3.91)	**2.42E−02**	0.725	0.673
Tumour stage	pT3 vs. pT2	5.18 (2.22–12.11)	**1.46E−04**	0.624	3.15 (1.29–7.70)	**1.20E−02**		
Progression score	Continuous	2.61 (1.63–4.18)	**7.08E−05**	0.672	2.09 (1.29–3.37)	**2.66E−03**		
Taylor (*n* = 107, 13 BCR)								
PSA	Continuous	1.04 (0.99–1.10)	9.32E−02	0.671	—	—	—	—
Margin status	Pos. vs. neg.	3.14 (1.02–9.69)	**4.66E−02**	0.653	4.32 (1.37–13.65)	**1.26E−02**	0.870	0.653
Gleason score	>7 vs. ≤7	8.66 (2.49–30.28)	**6.92E−04**	0.670	—	—		
Tumour stage	pT3 vs. pT2	2.83 (0.91–8.78)	7.25E−02	0.639	—	—		
Progression score	Continuous	5.66 (2.05–15.64)	**8.29E−04**	0.829	5.64 (2.11–15.07)	**5.63E−04**		
Long (*n* = 91, 40 BCR)								
PSA	Continuous	1.10 (1.07–1.13)	**1.61E−09**	0.719	1.09 (1.05–1.12)	**3.85E−07**		
Margin status	Pos. vs. neg.	3.29 (1.73–6.27)	**2.92E−04**	0.649	2.31 (1.16–4.61)	**1.67E−02**		
Gleason score	>7 vs. ≤7	2.71 (1.24–5.93)	**1.24E−02**	0.575	—	—		
Tumour stage	pT3 vs. pT2	1.90 (0.85–4.29)	1.20E−01	0.562	—	—		
Progression score	Continuous	2.33 (1.27–4.28)	**6.80E−03**	0.628	1.72 (0.98–3.04)	5.96E−02		

Seeding model								
		Univariate			Multivariate			
Variable	Characteristics	HR	*P*-value	*C*-index	HR	*P*-value	C-index	C-index*
TCGA (*n* = 405, 58 BCR)								
PSA	Continuous	1.03 (1.01–1.04)	**4.60E−03**	0.610	—	—	—	—
Margin status	Pos. vs. neg.	1.30 (0.77–2.19)	3.30E−01	0.519	—	—	—	—
Gleason score	>7 vs. ≤7	3.79 (2.10–6.84)	**9.38E−06**	0.642	—	—	—	—
Tumour stage	pT3 vs. pT2	5.18 (2.22–12.11)	**1.46E−04**	0.624	3.70 (1.55–8.84)	**3.17E−03**	0.722	0.624
Seeding score	Continuous	2.39 (1.81–3.16)	**1.05E−09**	0.705	2.01 (1.51–2.69)	**2.46E−06**		
Taylor (*n* = 107, 13 BCR)								
PSA	Continuous	1.04 (0.99–1.10)	9.32E−02	0.671	—	—	—	—
Margin status	Pos. vs. neg.	3.14 (1.02–9.69)	**4.66E−02**	0.653	4.77 (1.52–15.01)	**7.52E−03**	0.785	0.653
Gleason score	>7 vs. ≤7	8.69 (2.49–30.28)	**6.92E−04**	0.670	—	—		
Tumour stage	pT3 vs. pT2	2.83 (0.91–8.78)	7.25E−02	0.639	—	—		
Seeding score	Continuous	4.31 (1.94–9.57)	**3.37E−04**	0.777	4.85 (2.17–10.86)	**1.23E−04**		
Long (*n* = 91, 40 BCR)								
PSA	Continuous	1.10 (1.07–1.13)	**1.61E−09**	0.719	1.12 (1.08–1.16)	**3.15E−09**	0.807	0.719
Margin status	Pos. vs. neg.	3.29 (1.73–6.27)	**2.92E−04**	0.649	—	—		
Gleason score	>7 vs. ≤7	2.71 (1.24–5.93)	**1.24E−02**	0.575	—	—		
Tumour stage	pT3 vs. pT2	1.90 (0.85–4.29)	1.20E−01	0.562	—	—		
Seeding score	Continuous	2.79 (1.80–4.34)	**4.86E−06**	0.704	2.70 (1.78–4.10)	**3.27E−06**		

Significant *P* values (*P* *<* 0.05) are highlighted in bold

C-index = Harrell’s C-index for final model including progression model (top) or seeding model (bottom)

C-index* = Harrell’s C-index for final model excluding progression model (top) or seeding model  (bottom)

### Identification of the most-probable seeding focus

**Fig. 3:**
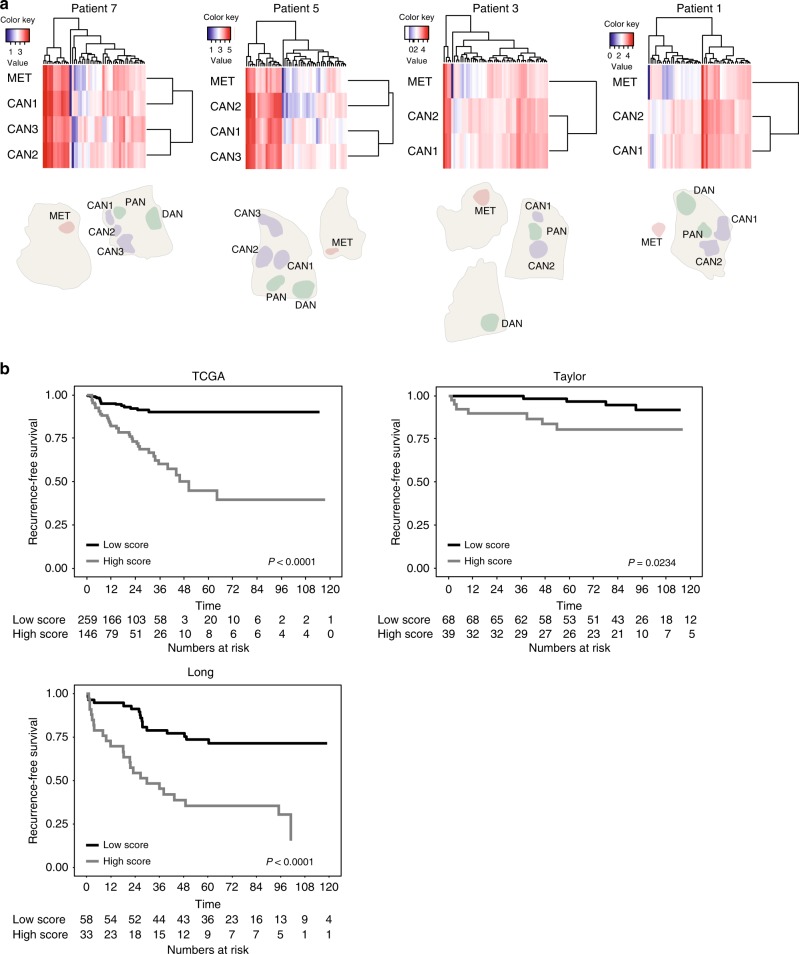
Identification of seeding foci. **a** Using clustering analysis, the most likely seeding focus was identified in two of the four patients (patient 7 and 5) with a minimum of two CAN samples and one matched MET sample. **b** Kaplan–Meier analysis of the seeding model in the TCGA, Taylor, and Long cohorts

For four of the ten patients in the discovery cohort (PT1, PT3, PT5 and PT7), multiple primary tumour samples as well as matched metastatic samples were analysed (Table 1 and Fig. 3a). We hypothesised that the primary tumour focus from which the metastasis originated could be determined based on transcriptional profile similarity. Using clustering analysis of the samples we could identify one likely seeding focus and two likely non-seeding foci for patient PT5 and patient PT7 (Fig. 3a). To identify transcripts that could be involved in the seeding of the metastatic sample, i.e. associated with increased aggressiveness, we compared the likely seeding focus versus the likely non-seeding focus/foci using differential gene-expression analysis. The differentially expressed transcripts between seeding foci (*n* = 2) and non-seeding foci (*n* = 4), included 49 transcripts (FDR < 0.05, 5 upregulated and 44 downregulated; Table S11). Using GSEA, five significantly enriched gene sets (FDR < 0.25) were identified in the seeding foci compared to non-seeding foci including, e.g. HALLMARK_ANDROGEN_RESPONSE and HALLMARK_HEDGEHOG_SIGNALING (Table S12), suggesting that these gene sets might be involved in the metastatic process.

Applying a cutoff value of FDR < 0.01 to the list of 49 deregulated transcripts, revealed a list of 20 transcripts (2 upregulated, 18 downregulated; Table S4). These transcripts were used to calculate a weighted seeding model for prognostic testing in each the three validation cohorts (Methods). The seeding model significantly predicted time to BCR independently of clinicopathological variables in uni- and multivariate Cox-regression analysis in TCGA (univariate: HR 2.39, *P* < 0.0001, multivariate: HR 2.01, *P* < 0.0001), Taylor (univariate: HR 4.31, *P* = 0.0003, multivariate: HR 4.85, *P* = 0.0001), and Long (univariate: HR 2.79, *P* < 0.0001, multivariate: HR 2.70, *P* < 0.0001) (Table 2). Using Kaplan–Meier analysis, the seeding model could successfully divide patients in high- and low risk groups to experience BCR in TCGA (*P* < 0.0001, log-rank test), Taylor (*P* = 0.0234, log-rank test), and in Long (*P* < 0.0001, log-rank test) (Fig. 3b). The cutoff was set using the fractions defined in the TCGA cohort. Moreover, a significantly higher seeding model score was associated with positive nodal status, higher Gleason score (>7 vs. ≤7), and advanced tumour stage (pT3 versus pT2) in TCGA, and higher Gleason score (>7 vs. ≤7) in Taylor (Wilcoxon rank-sum test; Figure S4B). The seeding model was not significantly associated with any clinicopathological variables in Long (Wilcoxon rank-sum test; Figure S4B).

In summary, the progression and seeding models developed from our relatively small cohort of 10 PC patients with matched primary tumour and metastatic tissue samples were shown to have prognostic potential in three independent RP cohorts, and could be relevant as prognostic markers to assess metastatic potential and/or risk of recurrence in early stage PC. Further validation is warranted.

## Discussion

One of the greatest hurdles in prostate cancer management is to accurately discern the aggressiveness of the individual cancer at the time of diagnosis. Many tumours remain indolent while some progress to advanced disease. Current clinicopathological parameters are lacking in prognostic accuracy, resulting in both overtreatment of indolent tumours and undertreatment of aggressive tumours. In the clinical management of prostate cancer patients, diagnostic needle biopsy samples are taken from the prostate, and assessed by a pathologist. However, due to the often multifocal origin of prostate cancer, it is still uncertain if all foci are represented, and to which extent the biopsy samples can predict prognosis. By transcriptomic profiling all tumour foci with matched metastases, results could plausibly enable the development of an aggressiveness gene signature representing the entire disease. Here, we present a study including a cohort of 10 hormone-naive PC patients with multiple cancer foci matched with metastatic samples, which enables a unique possibility to uncoil important markers of the metastatic process not confounded by drug treatment effects. Thus, in the study presented, we successfully generated RNA-Seq data from 57 different samples types (CAN, AN, MET, and LYMPH). We identified several differentially expressed transcripts between AN, CAN and MET samples, many of which have previously been associated with PC progression,^13,39–43^ thereby supporting the validity of our study. In addition, we identified two gene-expression-based models with strong prognostic potential using two different strategies. Firstly, genes that were downregulated from AN to CAN, and further downregulated from CAN to MET (in total 19 genes), were used to construct a progression model. Secondly, using stringent clustering criteria, we identified the most likely seeding focus in the primary tumour of two patients and developed a seeding model from the 20 most significantly differentially expressed genes between likely seeding- and likely non-seeding foci. Both models were significantly associated with more aggressive clinicopathological variables, and displayed strong prognostic potential in three independent RP cohorts (more than 650 patients in total) using univariate- and multivariate Cox-regression and Kaplan–Meier analysis.

During technical analysis of our sequencing pipeline, we observed some variations in mapping percentages. While the range was wider than expected, the mapping percentages below 30% for a fraction of samples, correspond to previous observation for PC FFPE samples with mapping percentages from 29 to 49% (Hedegaard et al.^44^). Archival FFPE tissue, which is often easily available in pathological archives, include samples with degraded RNA as input for RNA-Seq, which complicates novel analyses of PC related events such as gene fusions, chromoplexia and chromotrypsis. Future studies could aim to analyse, e.g. fresh-frozen tissue with high quality RNA.

Due to the high similarity observed between DAN and PAN samples, we collapsed these two sample groups into a single AN group. However, seven significantly differentially expressed transcripts were observed, and further investigation of these transcripts could provide novel information regarding differences in gene expression in a possible field effect depending on distance to the primary tumour focus. GSEA analysis revealed a negative enrichment of the p53 and Notch pathway in PAN samples, which is interesting due to their previous association with cancer-associated fibroblasts.^45^ Further studies on possible field effects, and the possible involvement of p53 and Notch signaling in normal adjacent tissue to cancer tissue, are reserved for future work including a larger sample set.

Some of the genes in the progression model have previously been studied. Interestingly, three studies with overlapping genes (*KRT5, KRT15, KRT23, KCTD14* and *RBFOX3*) were found, which represented different stages of PC development (CAN versus AN samples,^41^ CAN versus MET samples,^13^ and multiple stages of PC progression in a mouse-model^39^). In addition, these studies included fresh-frozen tissue samples as opposed to FFPE^13,41^ indicating a robustness of the identified candidates across different material types and study designs. MIR205HG was the most significantly downregulated transcript from AN to CAN and from CAN to MET in our cohort, which is in line with a previous study in PC reporting this miRNA as downregulated in the epithelial to mesenchymal transition by inhibiting ZEB2.^46^

In the discovery cohort, we managed to identify seeding foci in two out of four patients with multiple primary PC foci. The reasons for not identifying one in all four patients, could be due to e.g. possible variations in monoclonal versus polyclonal origins of the metastasis, as previously described.^5,7,47^ Additional variation in transcriptional profiles within the likely seeding focus could introduce another source of noise, as exemplified by Suh et al.,^48^ reporting intrafocal heterogeneity of ERG protein expression and gene fusion patterns in PC. Nevertheless, the likely seeding foci identified in our study gave rise to a seeding model, which showed high potential in predicting patients with high risk of BCR in three large independent RP patient cohorts including more than 650 PC patients. Furthermore, GSEA identified Hedgehog and androgen signaling as enriched gene sets in the likely seeding foci. These results implicate Hedgehog signaling in PC progression, an association which is previously described,^49–51^ but further studies are warranted to confirm this association.

There are some limitations to the present study. The discovery dataset was rather small, counting 59 samples from 10 PC patients. Nevertheless, the successful validation of both the progression model and the seeding model in three independent cohorts indicates that our novel marker panels are robust, despite the small discovery cohort. Previous studies^52^ have also shown that some prognostic candidate markers identified in small discovery cohorts can be successfully validated in larger cohorts, as was also the case in our current study. We note that the C-indices for our novel models ranged from 0.722 to 0.870. Thus, the observed C-indices for the progression model and the seeding model are comparable to those reported for the commercially available prognostic panels Decipher,^28,53–58^ OncotypeDx^23,59^ and Prolaris.^60–62^ Moreover, due to different expression profiling platforms in the external cohorts used in this study, it was impossible to thoroughly investigate all the 19 genes in the progression model or the 20 genes in the seeding model, since not all genes were present in all cohorts. In future studies, larger cohorts with a 100% gene overlap should be used when analyzing the seeding model and progression model as well as the genes included in these two models. Furthermore, the validation cohorts used in the present study had some different characteristics. The Long cohort was the smallest of the three validation cohorts, which may potentially explain at least in part, why our progression model was only borderline significant (*P* = 0.0596) in multivariate analysis in this cohort. Furthermore, the C-indices for Gleason score and tumour stage were only 0.575 and 0.562, respectively, for the Long cohort (Table 2), suggesting it may not be a fully representative RP cohort. Thus, further investigation using large independent cohorts with long follow-up and more clinically relevant end-points, such as distant metastases and/or PC-specific death, are needed to assess the clinical utility of the novel models presented here.

In conclusion, we identified several transcripts associated with the progression to metastatic PC. In addition, gene-expression-based models based on these genes could significantly divide patients in high and low risk to experience BCR in three external RP cohorts, implicating them as interesting clinical biomarker candidates. Additional large PC cohorts are needed to evaluate the clinical utility of the models.

## Electronic supplementary material

Supplementary information and Tables

Figure S1

Figure S2

Figure S3

## **Availability of data and material:**

Data from external cohorts can be found in the GEO database: Taylor (GSE21036), Long (GSE54460), and Grasso (GSE35988). TCGA data can be downloaded using the GDC data portal, NIH National Cancer Institute, USA^33^. RNA-Seq data for our discovery cohort is available from the authors upon request.
