# Supplementary material for: Exploring the transcriptome of hormone-naive multifocal prostate cancer and matched lymph node metastases
Source: Br J Cancer. 2018 Nov 19;119(12):1527–37. doi: 10.1038/s41416-018-0321-5 (PMC6288156; doi:10.1038/s41416-018-0321-5)
Supplement: Supplementary file 1 — Supplementary information and Tables [file 41416_2018_321_MOESM1_ESM.docx]

Supplementary information

Supplementary Methods, Tables and Supplementary Figures Legends

L. Schmidt *et al*:

*Exploring the transcriptome of hormone-naïve multifocal prostate cancer and matched lymph node metastases*

**SUPPLEMENTARY MATERIALS AND METHODS**

**Total RNA extraction, sequencing and analysis of the discovery cohort**

Prior to RNA extraction, deparaffination and laser-microdissection (LMD) was performed as previously described [1] (Figure S1A). Total RNA was extracted using the Allprep DNA/RNA FFPE Kit (Qiagen) and RNA concentrations were determined using the Quant-iT RiboGreen RNA assay kit (Invitrogen) or Qubit RNA BR Assay (Invitrogen). RNA size distribution was determined using the HT RNA High Sensitivity Assay for LabChip GX (PerkinElmer).

Automated sample preparations were performed on the Sciclone NGS Workstation (PerkinElmer): Total RNA (73-500 ng, mean: 453 ng) was depleted for rRNA using the Ribo-Zero Gold rRNA Removal Kit (Illumina), and the rRNA depleted RNA was purified using Agencourt RNAClean XP Kit (Beckman Coulter). Libraries were prepared with ScriptSeq Complete Gold Kit (Illumina) following the instructions for library preparation from severely fragmented RNA. The amplified libraries were purified using AMPure XP Beads (Beckman Coulter) and library size profiles were estimated using the DNA High Sensitivity Assay for LabChip GX (PerkinElmer). Libraries with a sharp peak at 150 base pair, i.e. not corresponding to the wanted product, were hereafter discarded (n=7) and low quality libraries (n=27), which contained a high amount of adaptor dimers, were bead purified an additional 1-2 times. Final library concentrations were measured using the KAPA Library Quantification Kit (Kapa Biosystems). Whole transcriptome, indexed, and paired-end (2x75bp) sequencing on a NextSeq 500 (Illumina) was performed for all sample libraries (10×DAN, 13×PAN, 23×CAN, 9×MET, 2×LYMPH) with an aim of 3x10^7^ mapped reads per samples. All sequencing data was included in further analysis, regardless of variations in the number of mapped reads between samples (see Table S1 for summary statistics of RNA-Seq data).

For data analysis, paired de-multiplexed fastq files were generated using bcf2fastq software (v2.20, Illumina) and the quality was checked using fastQC and fastqScreen. Adapter sequences were trimmed off using TrimGalore (v0.4.1) and Cutadapt (v1.9). The trimmed reads were mapped to the human genome (hg19) with the PAR1 and PAR2 regions on the Y chromosome masked out to better estimate the expression of pseudoautosomal genes on the X chromosome. TopHat2 (v2.1.1) [2] and Bowtie2 (v2.2.8) [3] were used without an annotation file. The CollectRnaSeqMetrics tool (Picard tools, v2.0.1) [4] was used to collect metrics about the alignments. Cufflinks (v2.2.1) [5] was used to quantitate the expression of all transcripts in the Gencode v19 gene model and raw reads were counted using htSeq (v0.6.1) [6]. Files with count-values of all transcripts were exported for further analysis in R [7]. Genes with two counts per million (CPM) or less, in three or more groups (DAN, PAN, CAN, MET, or LYMPH) where discarded, leaving 20,034 transcripts for further analysis. Heatmaps for clustering analysis were constructed using the heatmap.2 function in the ‘gplots’ packages in R [7]. Differential expression analyses was conducted in R using the ‘edgeR’ package [8].

**Supplementary Figure Legends**

**Figure S1. Workflow of preparation and overview of samples.** (A) Workflow for preparing samples. (B) HE stained sections of prostate and lymph node FFPE tissue from ten radical prostatectomy patients (PT1-PT10). Tumor and non-malignant areas are marked for laser microdissection (DAN: distant adjacent normal, PAN: proximal adjacent normal, CAN: primary tumor, MET: lymph node metastasis, LYMPH: non-malignant lymph node). Asterisks mark samples that did not yield sufficient library for RNA-seq.

**Figure S2. Principal component analysis and GSEA analysis.** (A) The gene set “TOMLINS_PROSTATE_CANCER_UP” is significantly enriched in CAN samples compared to AN samples.

**Figure S3. *Progression*- and *seeding model* scores are significantly higher in more aggressive prostate cancer tumors.** (A) *Progression model* scores are significantly higher in patients with BCR, higher Gleason score, positive node status and higher tumor stage in TCGA (top panel), higher in patients with BCR and higher Gleason score in the Taylor et al. cohort (middle panel), and higher in patients with BCR in Long et el. cohort (bottom panel). (B) *Seeding model* scores are significantly higher in patients with BCR, higher Gleason score, positive node status, higher tumor stage and higher preoperative PSA values in TCGA (top panel), higher in patients with BCR and higher Gleason score in the Taylor et al. cohort (middle panel), and higher in patients with BCR in the Long et al. cohort (bottom panel).

| **Table S1.** Summary statistics of RNA-Seq data | | | |
| --- | --- | --- | --- |
| **Sample** | **Reads** | **Mapped Reads** | **% Mapped Reads** |
| PT1_CAN_1 | 130,177,248 | 60,374,262 | 46.4 |
| PT1_CAN_2 | 91,998,490 | 46,159,257 | 50.2 |
| PT1_DAN | 90,699,708 | 33,800,130 | 37.3 |
| PT1_MET | 49,452,842 | 39,201,023 | 79.3 |
| PT1_PAN | 120,303,364 | 36,627,901 | 30.4 |
| PT2_CAN | 57,500,804 | 39,909,023 | 69.4 |
| PT2_DAN | 93,064,418 | 50,233,343 | 54.0 |
| PT2_LYMPH | 4,950,136 | 3,258,582 | 65.8 |
| PT2_MET | 160,356,738 | 81,085,743 | 50.6 |
| PT2_PAN | 82,035,884 | 52,542,245 | 64.0 |
| PT3_CAN_1 | 108,359,588 | 66,234,424 | 61.1 |
| PT3_CAN_2 | 127,598,746 | 65,635,150 | 51.4 |
| PT3_DAN | 61,509,720 | 46,843,862 | 76.2 |
| PT3_MET | 3,972,316 | 3,159,196 | 79.5 |
| PT3_PAN | 97,016,448 | 40,306,079 | 41.5 |
| PT4_CAN | 58,150,292 | 44,777,928 | 77.0 |
| PT4_DAN | 62,513,472 | 46,928,167 | 75.1 |
| PT4_LYMPH | 94,072,622 | 53,241,175 | 56.6 |
| PT4_MET_1 | 49,776,638 | 41,677,797 | 83.7 |
| PT4_MET_2 | 64,545,838 | 51,121,333 | 79.2 |
| PT4_MET_3 | 49,071,724 | 40,004,922 | 81.5 |
| PT4_PAN_1 | 99,387,622 | 57,758,921 | 58.1 |
| PT4_PAN_2 | 72,122,930 | 39,050,185 | 54.1 |
| PT5_CAN_1 | 96,770,482 | 46,409,475 | 48.0 |
| PT5_CAN_2 | 86,039,948 | 51,078,346 | 59.4 |
| PT5_CAN_3 | 99,869,798 | 32,892,631 | 32.9 |
| PT5_DAN | 110,391,716 | 29,934,136 | 27.1 |
| PT5_MET | 90,171,680 | 51,333,649 | 56.9 |
| PT5_PAN | 111,619,598 | 70,612,721 | 63.3 |
| PT6_CAN | 52,772,696 | 39,263,669 | 74.4 |
| PT6_DAN | 79,468,764 | 57,066,335 | 71.8 |
| PT6_MET | 95,028,478 | 51,061,593 | 53.7 |
| PT6_PAN | 73,494,188 | 55,018,533 | 74.9 |
| PT7_CAN_1 | 122,104,276 | 50,921,583 | 41.7 |
| PT7_CAN_2 | 143,252,540 | 47,991,062 | 33.5 |
| PT7_CAN_3 | 56,918,426 | 36,890,120 | 64.8 |
| PT7_DAN | 113,444,396 | 31,564,409 | 27.8 |
| PT7_MET | 109,378,232 | 31,478,618 | 28.8 |
| PT7_PAN | 103,293,664 | 65,575,188 | 63.5 |
| PT8_CAN_1 | 57,785,122 | 44,106,327 | 76.3 |
| PT8_CAN_2 | 76,191,760 | 52,882,736 | 69.4 |
| PT9_CAN_1 | 92,295,300 | 63,554,785 | 68.9 |
| PT9_CAN_2 | 49,286,890 | 32,126,058 | 65.2 |
| PT9_CAN_3 | 86,591,842 | 52,295,669 | 60.4 |
| PT9_CAN_4 | 156,340,026 | 54,278,371 | 34.7 |
| PT9_DAN | 85,449,142 | 36,476,316 | 42.7 |
| PT9_PAN | 202,021,002 | 66,025,192 | 32.7 |
| PT10_CAN_1 | 61,876,972 | 49,132,058 | 79.4 |
| PT10_CAN_2 | 49,117,400 | 41,205,007 | 83.9 |
| PT10_CAN_3 | 87,746,264 | 39,505,351 | 45.0 |
| PT10_CAN_4 | 133,289,160 | 65,403,993 | 49.1 |
| PT10_DAN_1 | 110,377,036 | 61,684,584 | 55.9 |
| PT10_DAN_2 | 99,241,908 | 36,711,405 | 37.0 |
| PT10_PAN_1 | 73,822,200 | 43,947,940 | 59.5 |
| PT10_PAN_2 | 102,047,778 | 51,263,084 | 50.2 |
| PT10_PAN_3 | 88,555,106 | 47,412,470 | 53.5 |
| PT10_PAN_4 | 96,523,360 | 43,113,074 | 44.7 |

| **Table S2**. Clinicopathological characteristics of the three cohorts used for assessing prognostic potential of the *progression*- and *seeding models* | | | |
| --- | --- | --- | --- |
|  |  |  |  |
|  | **TCGA PRAD Cohort** | **Taylor Cohort** | **Long Cohort** |
| **Samples** | RP (n = 477) | RP (n = 107) | RP (n = 91) |
| **Median Age, years (range)** | 61 (41 - 78) | 57.5 (37.3 - 71.2) | 61.4 (43 - 78) |
| **Preoperative PSA, n (%)** |  |  |  |
| < 10 ng/mL | 314 (65.8) | 86 (80.4) | 61 (67.0) |
| ≥ 10 ng/mL | 148 (31.0) | 21 (19.6) | 27 (29.7) |
| Unknown | 15 (3.2) | 0 (0.0) | 3 (3.3) |
| Median PSA, ng/mL (range) | 7.5 (0.7 - 107) | 5.8 (1.2 - 46.4) | 7.2 (1.8 - 72.6) |
| **Pathological T-stage, n (%)** |  |  |  |
| pT1a-c | 0 (0.0) | 0 (0.0) | 11 (12.1) |
| pT2a-c | 184 (38.6) | 75 (70.1) | 64 (70.3) |
| pT3a-c | 276 (57.9) | 28 (26.2) | 14 (15.4) |
| pT4 | 10 (2.1) | 4 (3.7) | 1 (1.1) |
| Unknown | 7 (1.4) | 0 (0.0) | 1 (1.1) |
| **Gleason score, n (%)** |  |  |  |
| ≤ 7 | 285 (59.7) | 101 (94.4) | 80 (87.9) |
| > 7 | 192 (40.3) | 6 (5.6) | 11 (12.1) |
| Unknown | 0 (0.0) | 0 (0.0) | 0 (0.0) |
| **Surgical margin status, n (%)** |  |  |  |
| Negative | 305 (64.0) | 85 (79.4) | 54 (59.3) |
| Positive | 137 (28.7) | 22 (20.6) | 32 (35.2) |
| Unknown | 35 (7.3) | 0 (0.0) | 5 (5.5) |
| **Nodal status, n (%)** |  |  |  |
| Negative | 314 (65.8) | 83 (77.6) | NA |
| Positive | 73 (15.3) | 3 (2.8) | NA |
| Unknown | 90 (18.9) | 21 (19.6) | NA |
| **Recurrence status, n (%)** |  |  |  |
| No recurrence | 351 (73.6) | 94 (87.9) | 51 (56.0) |
| Recurrence | 58 (12.2) | 13 (12.1) | 40 (44.0) |
| Unknown | 68 (14.2) | 0 (0.0) | 0 (0.0) |
| **Median follow-up time, months (range)** | 15 (<1 - 151.3) | 88.9 (1.6 - 222.5) | 69.0 (<1 - 180.6) |

| **Table S3.** Genes in *progression signature* (n=19). (FDR<0.05 and sorted on Fc) | | | | | | | | |
| --- | --- | --- | --- | --- | --- | --- | --- | --- |
|  |  | **AN - CAN** | | |  | **CAN - MET** | | |
| **Gene id** | **Gene name** | **logFC** | **P value** | **FDR** |  | **logFC** | **P value** | **FDR** |
| ENSG00000101951.12 | PAGE4 | -2.991 | 0.000 | 0.000 |  | -6.997 | 0.000 | 0.000 |
| ENSG00000151364.11 | KCTD14 | -2.189 | 0.002 | 0.044 |  | -6.311 | 0.000 | 0.000 |
| ENSG00000250423.2 | KIAA1210 | -2.398 | 0.000 | 0.001 |  | -5.417 | 0.000 | 0.000 |
| ENSG00000108244.12 | KRT23 | -3.146 | 0.000 | 0.001 |  | -5.303 | 0.000 | 0.004 |
| ENSG00000163810.7 | TGM4 | -4.147 | 0.000 | 0.003 |  | -4.798 | 0.001 | 0.030 |
| ENSG00000261116.1 | RP3-523K23.2 | -4.021 | 0.000 | 0.002 |  | -4.690 | 0.001 | 0.018 |
| ENSG00000186081.7 | KRT5 | -3.382 | 0.000 | 0.000 |  | -4.608 | 0.000 | 0.000 |
| ENSG00000174403.11 | C20orf166-AS1 | -2.364 | 0.000 | 0.009 |  | -4.573 | 0.000 | 0.002 |
| ENSG00000230937.3 | MIR205HG | -3.882 | 0.000 | 0.000 |  | -4.492 | 0.000 | 0.002 |
| ENSG00000266221.1 | RP11-746E8.1 | -2.910 | 0.000 | 0.009 |  | -4.455 | 0.001 | 0.028 |
| ENSG00000198732.6 | SMOC1 | -2.161 | 0.000 | 0.000 |  | -4.187 | 0.000 | 0.000 |
| ENSG00000197467.7 | COL13A1 | -2.008 | 0.000 | 0.001 |  | -3.997 | 0.000 | 0.000 |
| ENSG00000167281.13 | RBFOX3 | -2.151 | 0.000 | 0.012 |  | -3.869 | 0.002 | 0.031 |
| ENSG00000225504.3 | RP11-160A10.2 | -2.291 | 0.001 | 0.027 |  | -3.645 | 0.002 | 0.038 |
| ENSG00000168143.8 | FAM83B | -3.076 | 0.000 | 0.000 |  | -3.350 | 0.002 | 0.033 |
| ENSG00000125848.9 | FLRT3 | -2.263 | 0.000 | 0.004 |  | -3.174 | 0.000 | 0.011 |
| ENSG00000145113.17 | MUC4 | -2.991 | 0.000 | 0.001 |  | -2.962 | 0.001 | 0.023 |
| ENSG00000171346.9 | KRT15 | -3.333 | 0.000 | 0.000 |  | -2.618 | 0.000 | 0.008 |
| ENSG00000184313.14 | HEATR8 | -2.020 | 0.000 | 0.000 |  | -2.556 | 0.000 | 0.007 |
| logFC = log2 fold change between the groups, FDR = False Discovery Rate | | | | | | | | |
|  |  |  |  |  |  |  |  |  |

| **Table S4.** *Seeding model* genes in 10-patient set | | | | | |  |
| --- | --- | --- | --- | --- | --- | --- |
| **Seeding model genes** | | | | | | |
| *ENSMBL* | *Gene ID* | *logFC* | *logCPM* | *LR* | *P value* | *FDR* |
| ENSG00000132872.6 | SYT4 | -5.73 | 6.65 | 32.2 | 1.39E-08 | 2.15E-04 |
| ENSG00000148513.12 | ANKRD30A | 6.73 | 7.05 | 30.46 | 3.41E-08 | 2.64E-04 |
| ENSG00000173200.8 | PARP15 | -6.43 | 4.71 | 28.59 | 8.95E-08 | 4.61E-04 |
| ENSG00000267002.1 | RP11.242D8.1 | -9.4 | 3.26 | 27.82 | 1.33E-07 | 5.13E-04 |
| ENSG00000248441.2 | CTD-2536I1.1 | 7.57 | 2.42 | 26.67 | 2.41E-07 | 5.69E-04 |
| ENSG00000265625.1 | RP11-68I3.11 | -8.84 | 3.26 | 26.37 | 2.82E-07 | 5.69E-04 |
| ENSG00000111358.8 | GTF2H3 | -9.22 | 3.17 | 26.28 | 2.95E-07 | 5.69E-04 |
| ENSG00000204394.8 | VARS | -9.45 | 3.12 | 27.16 | 1.88E-07 | 5.69E-04 |
| ENSG00000168685.10 | IL7R | -3.58 | 5.69 | 25.92 | 3.56E-07 | 6.12E-04 |
| ENSG00000070190.8 | DAPP1 | -6.74 | 3.51 | 24.92 | 5.99E-07 | 9.25E-04 |
| ENSG00000162631.13 | NTNG1 | -3.71 | 6.07 | 23.64 | 1.16E-06 | 1.63E-03 |
| ENSG00000185404.12 | SP140L | -7.13 | 3.34 | 23.42 | 1.30E-06 | 1.67E-03 |
| ENSG00000221817.3 | RP11-137L10.6 | -8.95 | 3.12 | 22.27 | 2.37E-06 | 2.81E-03 |
| ENSG00000054219.9 | LY75 | -8.53 | 2.9 | 21.98 | 2.75E-06 | 3.04E-03 |
| ENSG00000153896.12 | ZNF599 | -8.87 | 3.07 | 21.81 | 3.01E-06 | 3.10E-03 |
| ENSG00000248329.1 | RP11-366M4.3 | -9.09 | 2.97 | 20.86 | 4.94E-06 | 4.77E-03 |
| ENSG00000136960.7 | ENPP2 | -5.21 | 3.78 | 20.32 | 6.53E-06 | 5.94E-03 |
| ENSG00000164122.4 | ASB5 | -10.56 | 3.87 | 19.98 | 7.83E-06 | 6.72E-03 |
| ENSG00000262714.1 | RP11-44F14.8 | -7.36 | 3.17 | 19.88 | 8.27E-06 | 6.72E-03 |
| ENSG00000246339.3 | EXTL3-AS1 | -8.6 | 2.64 | 19.7 | 9.05E-06 | 6.99E-03 |
| logFC = log2 fold change between the groups, logCPM = the average log2-counts-per-million, LR = likelihood ratio statistics, *P* value = the two-sided P value, FDR = False discovery rate | | | | | | |

| **Table S5.** Differentially expressed transcripts PAN versus DAN (FDR <0.05) | | | | | |  |
| --- | --- | --- | --- | --- | --- | --- |
| *ENS_ID* | *GENE ID* | *logFC* | *logCPM* | *LR* | *P value* | *FDR* |
| ENSG00000236678.2 | LINC00347 | 5.00 | -1.14 | 36.33 | 1.66E-09 | **3.87E-05** |
| ENSG00000177992.9 | SPATA31E1 | -4.96 | -0.60 | 23.21 | 1.45E-06 | **1.69E-02** |
| ENSG00000231150.1 | RP1-207H1.3 | -5.60 | 3.50 | 21.66 | 3.25E-06 | **1.94E-02** |
| ENSG00000266141.1 | MIR2909 | -5.94 | 0.66 | 21.61 | 3.34E-06 | **1.94E-02** |
| ENSG00000234084.1 | RP3-388E23.2 | 5.48 | 0.45 | 21.01 | 4.56E-06 | **2.12E-02** |
| ENSG00000235326.1 | RP11-509J21.3 | -5.05 | 1.01 | 19.16 | 1.20E-05 | **4.67E-02** |
| ENSG00000260729.1 | RP11-106M3.2 | -6.03 | -0.18 | 18.80 | 1.45E-05 | **4.83E-02** |

logFC = log2 fold change between the groups, logCPM = the average log2-counts-per-million, LR = likelihood ratio statistics,
*P* value = the two-sided *P* value, FDR = False discovery rate

| **Table S6.** GSEA on PAN versus DAN samples | | | | | | |
| --- | --- | --- | --- | --- | --- | --- |
| Enriched in PAN versus DAN | | | | | | |
| **NAME** | **SIZE** | **ES** | **NES** | **NOM p-val** | **FDR q-val** | **FWER p-val** |
| HALLMARK_P53_PATHWAY | 149 | -0.46 | -1.57 | 0.00E+00 | 3.30E-02 | 6.60E-02 |
| HALLMARK_NOTCH_SIGNALING | 27 | -0.53 | -1.53 | 1.27E-02 | 3.81E-02 | 1.11E-01 |
| HALLMARK_PROTEIN_SECRETION | 92 | -0.43 | -1.43 | 7.02E-03 | 1.06E-01 | 3.66E-01 |
| PAN = proximal adjacent normal, DAN = distant adjacent normal. Size = number of genes in gene set, ES = enrichment score, NES = normalized ES, NOM P value = the statistical significance of the enrichment score, FDR q-value = the estimated probability that the normalized enrichment score represents a false positive finding, FDR = False discovery rate, FWER P value = familywise-error rate, a more conservatively estimated probability that the NES represents a false positive finding. | | | | | | |
|  |  |  |  |  |  |  |

**Table S7.** Differentially expressed transcript in Cancer tissue samples (CAN) versus Normal Adjacent tissue samples (AN)

| **Upregulated in CAN** | | | | | | | |  |  |
| --- | --- | --- | --- | --- | --- | --- | --- | --- | --- |
| *ENS ID* | *GENE ID* | *logFC* | *logCPM* | *LR* | *P value* | *FDR* | *Previous studies* |  |  |
| ENSG00000175018.8 | TEX36 | 4.87 | -0.98 | 71.39 | 2.93E-17 | 3.41E-13 |  |  |  |
| ENSG00000225937.1 | **PCA3** | 4.44 | 8.03 | 57.81 | 2.89E-14 | 1.12E-10 | [9, 10] |  |  |
| ENSG00000135052.11 | **GOLM1** | 2.04 | 8.84 | 51.47 | 7.26E-13 | 2.11E-09 | [11, 12] |  |  |
| ENSG00000242110.3 | **AMACR** | 4.47 | 5.79 | 51.68 | 6.55E-13 | 2.11E-09 | [10, 13, 14] |  |  |
| ENSG00000166206.8 | GABRB3 | 2.55 | 5.67 | 48.53 | 3.26E-12 | 7.58E-09 |  |  |  |
| ENSG00000143797.7 | MBOAT2 | 2.08 | 7.71 | 45.77 | 1.33E-11 | 2.21E-08 |  |  |  |
| ENSG00000168903.8 | BTNL3 | 4.33 | -0.87 | 45.90 | 1.25E-11 | 2.21E-08 |  |  |  |
| ENSG00000095627.5 | **TDRD1** | 3.82 | 5.04 | 44.96 | 2.01E-11 | 3.12E-08 | [15] |  |  |
| ENSG00000133019.7 | **CHRM3** | 2.90 | 5.43 | 42.13 | 8.54E-11 | 9.38E-08 | [16] |  |  |
| ENSG00000248461.1 | LINC02119 | 4.00 | -0.65 | 42.10 | 8.68E-11 | 9.38E-08 |  |  |  |
| ENSG00000187398.7 | **LUZP2** | 3.08 | 6.18 | 37.97 | 7.17E-10 | 5.39E-07 | [17, 18] |  |  |
| ENSG00000157554.14 | **ERG** | 2.43 | 7.89 | 37.39 | 9.65E-10 | 6.81E-07 | [19, 20] |  |  |
| ENSG00000211689.2 | TRGC1 | 2.51 | 7.67 | 36.74 | 1.35E-09 | 8.62E-07 |  |  |  |
| ENSG00000237796.1 | RP11-789G22.1 | 5.28 | 0.14 | 36.66 | 1.41E-09 | 8.62E-07 |  |  |  |
| ENSG00000188848.11 | BEND4 | 2.05 | 6.62 | 34.72 | 3.81E-09 | 1.97E-06 |  |  |  |
| ENSG00000132932.11 | ATP8A2 | 4.15 | 3.89 | 34.46 | 4.36E-09 | 2.16E-06 |  |  |  |
| ENSG00000144355.10 | **DLX** | 5.08 | 2.77 | 34.08 | 5.29E-09 | 2.56E-06 | [18, 21, 22] |  |  |
| ENSG00000263765.1 | [LOC105372059](http://www.genecards.org/cgi-bin/carddisp.pl?gene=LOC105372059) | 3.50 | -0.90 | 33.82 | 6.04E-09 | 2.87E-06 |  |  |  |
| ENSG00000096006.7 | **CRISP3** | 4.74 | 7.11 | 32.88 | 9.81E-09 | 4.01E-06 | [10, 23, 24] |  |  |
| ENSG00000197757.7 | HXC6 | 3.49 | 3.77 | 32.76 | 1.04E-08 | 4.12E-06 |  |  |  |
| **Downregulated in CAN** | | | | | | | |  |  |
| *ENS ID* | *GENE ID* | *logFC* | *logCPM* | *LR* | *P value* | *FDR* | *Previous studies* |  |  |
| ENSG00000141469.12 | **SLC14A1** | -3.50 | 7.20 | 60.19 | 8.63E-15 | 5.02E-11 | [10] |  |  |
| ENSG00000152784.9 | PRDM8 | -2.67 | 4.93 | 59.25 | 1.39E-14 | 6.48E-11 |  |  |  |
| ENSG00000165078.7 | CPA6 | -4.63 | 3.09 | 47.62 | 5.19E-12 | 1.10E-08 |  |  |  |
| ENSG00000073282.7 | **TP63** | -3.26 | 5.88 | 43.00 | 5.47E-11 | 7.08E-08 | [25-27] |  |  |
| ENSG00000171346.9 | **KRT15** | -3.33 | 4.87 | 42.86 | 5.88E-11 | 7.20E-08 | [28] |  |  |
| ENSG00000170477.8 | KRT4 | -5.45 | 0.50 | 42.06 | 8.86E-11 | 9.38E-08 |  |  |  |
| ENSG00000186081.7 | **KRT5** | -3.38 | 5.46 | 36.68 | 1.39E-09 | 8.62E-07 | [28] |  |  |
| ENSG00000106258.9 | **CYP3A5** | -3.19 | 4.44 | 35.97 | 2.01E-09 | 1.11E-06 | [29] |  |  |
| ENSG00000230937.3 | **MIR205HG** | -3.88 | 6.33 | 33.45 | 7.30E-09 | 3.27E-06 | [30] |  |  |
| ENSG00000137699.10 | **TRIM29** | -3.95 | 4.50 | 33.19 | 8.36E-09 | 3.54E-06 | [31] |  |  |
| ENSG00000167165.12 | CD6 | -4.72 | -0.08 | 32.07 | 1.48E-08 | 5.67E-06 |  |  |  |
| ENSG00000267164.1 | AC005336.1 | -4.71 | 3.53 | 30.58 | 3.21E-08 | 1.08E-05 |  |  |  |
| ENSG00000101951.12 | **PAGE4** | -2.99 | 3.12 | 30.15 | 4.00E-08 | 1.27E-05 | [32] |  |  |
| ENSG00000197565.11 | **COL4A6** | -2.24 | 4.70 | 29.70 | 5.04E-08 | 1.51E-05 | [33, 34] |  |  |
| ENSG00000100285.9 | **NEFH** | -3.14 | 7.48 | 28.33 | 1.02E-07 | 2.81E-05 | [35] |  |  |
| ENSG00000141622.8 | **RNF165** | -2.42 | 4.00 | 28.26 | 1.06E-07 | 2.84E-05 | [36] |  |  |
| ENSG00000198732.6 | **SMOC1** | -2.16 | 5.27 | 27.41 | 1.64E-07 | 3.98E-05 | [37] |  |  |
| ENSG00000254951.2 | RP11-494M8.4 | -3.21 | 2.98 | 26.87 | 2.17E-07 | 4.97E-05 |  |  |  |
| ENSG00000100842.8 | **EFS** | -2.27 | 4.19 | 26.73 | 2.34E-07 | 5.19E-05 | [38] |  |  |
| ENSG00000250786.1 | SNHG18 | -3.03 | 2.99 | 26.23 | 3.03E-07 | 6.42E-05 |  |  |  |

logFC = log2 fold change between the groups, logCPM = the average log2-counts-per-million, LR = likelihood ratio statistics, *P* value = the two-sided
*P* value, FDR = False discovery rate. Genes underlined and marked in bold have previously been studied in prostate cancer.

| **Table S8.** GSEA on Cancer tissue samples (CAN) versus Normal Adjacent tissue samples (AN) (FDR <0.25) | | | | | | |
| --- | --- | --- | --- | --- | --- | --- |
| **Enriched in CAN versus AN** | | | | | | |
| *NAME* | *SIZE* | *ES* | *NES* | *NOM P value* | *FDR q-value* | *FWER P value* |
| HALLMARK_ANDROGEN_RESPONSE | 97 | 0.49 | 2.41 | 0.00E+00 | 0.00E+00 | 0.00E+00 |
| HALLMARK_MYC_TARGETS_V1 | 184 | 0.36 | 2.00 | 0.00E+00 | 7.53E-04 | 1.00E-03 |
| **Enriched in AN versus CAN** | | | | | | |
| *NAME* | *SIZE* | *ES* | *NES* | *NOM P value* | *FDR q-value* | *FWER P value* |
| HALLMARK_MYOGENESIS | 118.00 | -0.56 | -2.12 | 0.00E+00 | 0.00E+00 | 0.00E+00 |
| HALLMARK_APICAL_JUNCTION | 140.00 | -0.48 | -1.85 | 0.00E+00 | 8.22E-04 | 3.00E-03 |
| HALLMARK_EPITHELIAL_MESENCHYMAL_TRANSITION | 148.00 | -0.49 | -1.93 | 0.00E+00 | 8.32E-04 | 2.00E-03 |
| HALLMARK_COAGULATION | 67.00 | -0.49 | -1.74 | 0.00E+00 | 3.67E-03 | 2.00E-02 |
| HALLMARK_APICAL_SURFACE | 28.00 | -0.57 | -1.76 | 1.26E-03 | 4.36E-03 | 1.90E-02 |
| HALLMARK_UV_RESPONSE_DN | 127.00 | -0.44 | -1.70 | 0.00E+00 | 5.12E-03 | 3.50E-02 |
| HALLMARK_APOPTOSIS | 133.00 | -0.42 | -1.62 | 0.00E+00 | 1.19E-02 | 9.30E-02 |
| HALLMARK_HEDGEHOG_SIGNALING | 20.00 | -0.57 | -1.60 | 1.56E-02 | 1.34E-02 | 1.20E-01 |
| HALLMARK_WNT_BETA_CATENIN_SIGNALING | 28.00 | -0.49 | -1.48 | 3.41E-02 | 4.47E-02 | 4.02E-01 |
| HALLMARK_ESTROGEN_RESPONSE_EARLY | 136.00 | -0.38 | -1.49 | 2.14E-03 | 4.48E-02 | 3.69E-01 |
|  |  |  |  |  |  |  |

Size = number of genes in gene set, ES = enrichment score, NES = normalized ES, NOM *P* value = the statistical significance of the enrichment score, FDR q-value = the estimated probability that the normalized enrichment score represents a false positive finding, FDR = False discovery rate, FWER *P* value = familywise-error rate, a more conservatively estimated probability that the NES represents a false positive finding.

| **Table S9.** Differentially expressed transcript in Metastatic tissue samples (MET) versus Cancer tissue samples (CAN) (top 20) | | | | | | | |  |
| --- | --- | --- | --- | --- | --- | --- | --- | --- |
| **Upregulated in MET** | | | | | | | |  |
| *ENS ID* | *GENE ID* | *logFC* | *logCPM* | *LR* | *P value* | *FDR* | *Previous studies* |  |
| ENSG00000137077.3 | CCL21 | 6.86 | 4.64 | 70.27 | 5.16E-17 | 3.01E-13 |  |  |
| ENSG00000162777.12 | DENND2D | 2.66 | 5.49 | 68.07 | 1.58E-16 | 6.12E-13 |  |  |
| ENSG00000104894.7 | CD37 | 4.20 | 4.83 | 62.39 | 2.82E-15 | 6.57E-12 |  |  |
| ENSG00000175463.7 | TBC1D10C | 4.11 | 3.42 | 52.08 | 5.34E-13 | 7.54E-10 |  |  |
| ENSG00000135905.13 | DOCK10 | 2.21 | 6.13 | 51.70 | 6.45E-13 | 8.35E-10 |  |  |
| ENSG00000072786.7 | STK10 | 2.15 | 4.85 | 48.54 | 3.24E-12 | 3.02E-09 |  |  |
| ENSG00000150636.11 | CCDC102B | 3.49 | 4.24 | 47.46 | 5.62E-12 | 4.85E-09 |  |  |
| ENSG00000242574.3 | HLA-DMB-006 | 2.28 | 6.22 | 44.82 | 2.16E-11 | 1.57E-08 |  |  |
| ENSG00000137841.7 | **PLCB2** | 3.05 | 4.25 | 43.70 | 3.83E-11 | 2.70E-08 | [39] |  |
| ENSG00000179583.13 | CIITA | 2.44 | 6.53 | 43.56 | 4.11E-11 | 2.81E-08 |  |  |
| ENSG00000081059.15 | **TCF7** | 2.85 | 5.34 | 43.46 | 4.34E-11 | 2.81E-08 | [40] |  |
| ENSG00000081237.14 | PTPRC | 2.17 | 7.53 | 43.10 | 5.20E-11 | 3.27E-08 |  |  |
| ENSG00000012124.10 | CD22 | 4.67 | 4.70 | 42.98 | 5.52E-11 | 3.38E-08 |  |  |
| ENSG00000196092.8 | PAX5 | 5.37 | 4.14 | 42.07 | 8.79E-11 | 4.90E-08 |  |  |
| ENSG00000167483.13 | FAM129C | 5.68 | 4.04 | 41.97 | 9.26E-11 | 4.90E-08 |  |  |
| ENSG00000146192.10 | FGD2 | 2.62 | 4.70 | 41.49 | 1.19E-10 | 6.00E-08 |  |  |
| ENSG00000166211.6 | SPIC | 5.88 | -0.58 | 41.23 | 1.35E-10 | 6.69E-08 |  |  |
| ENSG00000110777.7 | [POU2AF1](http://www.genecards.org/cgi-bin/carddisp.pl?gene=LOC105372059) | 4.06 | 4.24 | 40.90 | 1.60E-10 | 7.61E-08 |  |  |
| ENSG00000068831.14 | RASGRP2 | 3.41 | 4.26 | 37.72 | 8.16E-10 | 3.28E-07 |  |  |
| ENSG00000156738.13 | MS4A1 | 5.87 | 6.14 | 37.57 | 8.81E-10 | 3.48E-07 |  |  |
| **Downregulated in MET** | | | | | | | |  |
| *ENS ID* | *GENE ID* | *logFC* | *logCPM* | *LR* | *P value* | *FDR* | *Previous studies* |  |
| ENSG00000211448.7 | **DIO2** | -4.85 | 6.84 | 115.49 | 6.15E-27 | 1.43E-22 | [41] |  |
| ENSG00000164161.5 | HHIP | -5.61 | 6.63 | 92.91 | 5.48E-22 | 6.39E-18 |  |  |
| ENSG00000130176.3 | **CNN1** | -6.12 | 7.07 | 88.42 | 5.30E-21 | 4.12E-17 | [42-44] |  |
| ENSG00000175084.7 | DES | -5.88 | 8.21 | 69.30 | 8.45E-17 | 3.93E-13 |  |  |
| ENSG00000133392.11 | **MYH11** | -4.48 | 11.77 | 67.59 | 2.01E-16 | 6.70E-13 | [43] |  |
| ENSG00000183036.6 | **PCP4** | -6.89 | 6.09 | 64.00 | 1.25E-15 | 3.63E-12 | [44] |  |
| ENSG00000214548.8 | **MEG3** | -3.26 | 8.12 | 63.63 | 1.50E-15 | 3.88E-12 | [45] |  |
| ENSG00000159167.7 | STC1 | -3.57 | 5.17 | 54.23 | 1.78E-13 | 3.77E-10 |  |  |
| ENSG00000049319.2 | **SRD5A2** | -6.73 | 5.82 | 53.75 | 2.27E-13 | 4.07E-10 | [46] |  |
| ENSG00000196104.6 | **SPOCK3** | -6.13 | 6.86 | 53.86 | 2.15E-13 | 4.07E-10 | [47] |  |
| ENSG00000125868.10 | DSTN | -2.10 | 8.31 | 52.77 | 3.75E-13 | 6.24E-10 |  |  |
| ENSG00000016082.10 | ISL1 | -8.52 | 3.16 | 52.31 | 4.74E-13 | 7.36E-10 |  |  |
| ENSG00000163017.9 | **ACTG2** | -5.86 | 8.10 | 52.02 | 5.51E-13 | 7.54E-10 | [43, 44] |  |
| ENSG00000101335.5 | **MYL9** | -3.72 | 7.79 | 50.56 | 1.15E-12 | 1.34E-09 | [44] |  |
| ENSG00000101825.6 | MXRA5 | -3.68 | 6.63 | 50.62 | 1.12E-12 | 1.34E-09 |  |  |
| ENSG00000162772.12 | **ATF3** | -4.51 | 7.82 | 50.31 | 1.31E-12 | 1.40E-09 | [48, 49] |  |
| ENSG00000095637.13 | **SORBS1** | -3.09 | 8.85 | 50.30 | 1.32E-12 | 1.40E-09 | [50] |  |
| ENSG00000163171.6 | CDC42EP3 | -2.12 | 7.88 | 49.75 | 1.75E-12 | 1.77E-09 |  |  |
| ENSG00000123358.14 | **NR4A1** | -3.05 | 8.77 | 49.08 | 2.46E-12 | 2.38E-09 | [51] |  |
| ENSG00000154553.8 | PDLIM3 | -3.41 | 7.63 | 46.97 | 7.21E-12 | 5.99E-09 |  |  |

logFC = log2 fold change between the groups, logCPM = the average log2-counts-per-million, LR = likelihood ratio statistics, *P* value = the two-sided *P* value, FDR = False discovery rate. Genes underlined and marked in bold have previously been studied in prostate cancer

| **Table S10.** Downregulated transcripts overlapping with GSEA curated gene sets (modified output from online analysis) | | | | | |
| --- | --- | --- | --- | --- | --- |
| **Number of genes in gene set** | **Description** | **Overlapping genes** | ***P* value** | **FDR q-value** | **Reference** |
| 481 | Genes downregulated in prostate cancer samples. | 7 (*KRT15, KRT5, KRT23, COL13A1, FLRT3, MUC4, SMOC1*) | 4.24E-11 | 2.01E-07 | Liu et al.^[52]^ |
| 91 | Genes downregulated in lobular carcinoma vs normal ductal breast cells. | 4 (*KRT15, KRT5, KRT23, KCTD14*) | 1.42E-08 | 3.35E-05 | Turashvili et al.^[53]^ |
| 306 | Genes downregulated in metastatic tumors from the whole panel of patients with prostate cancer. | 4 (*KRT15, KRT5, COL13A1, RBFOX3*) | 1.83E-06 | 2.22E-03 | Chandran et al.^[28]^ |
| 308 | Genes downregulated during prostate cancer progression in the JOCK1 model due to inducible activation of FGFR1 [GeneID=2260] gene in prostate. | 4 (*KRT15, KRT5, FLRT3, RBFOX3*) | 1.88E-06 | 2.22E-03 | Acevedo et al.^[54]^ |
| 198 | Genes downregulated in ductal carcinoma vs normal ductal breast cells. | 3 (*KRT15, KRT5, KRT23*) | 2.77E-05 | 2.62E-02 | Turashvili et al.^[53]^ |
| 648 | Genes upregulated in basal subtype of breast cancer samples. | 4 (*KRT15, KRT5, KRT23, KCTD14)* | 3.50E-05 | 2.76E-02 | Smid et al.^[55]^ |
|  |  |  |  |  |  |
| 258 | Genes downregulated in metastases from malignant melanoma compared to the primary tumours. | 3 (*KRT15, KRT5, KRT23*) | 6.08E-05 | 4.11E-02 | Jaeger et al.^[56]^ |

| **Table S11.** Differentially expressed transcript in Seeding foci (n=2) versus non-Seeding foci (n=4) (FDR <0.05) | | | | | | |
| --- | --- | --- | --- | --- | --- | --- |
| **Upregulated in Seeding** | | | | | | |
| *ENS ID* | *GENE ID* | *logFC* | *logCPM* | *LR* | *PValue* | *FDR* |
| ENSG00000248441.2 | LINC01197 | 7.57 | 2.42 | 26.67 | 2.41E-07 | 5.69E-04 |
| ENSG00000148513.12 | ANKRD30A | 6.73 | 7.05 | 30.46 | 3.41E-08 | 2.64E-04 |
| ENSG00000128652.6 | HOXD3 | 4.10 | 3.87 | 16.81 | 4.13E-05 | 2.19E-02 |
| ENSG00000139970.12 | RTN1 | 2.69 | 5.28 | 16.66 | 4.46E-05 | 2.19E-02 |
| ENSG00000251733.1 | SCARNA8 | 2.63 | 6.27 | 15.88 | 6.77E-05 | 2.64E-02 |
| **Downregulated in Seeding** | | | | | | |
| *ENS ID* | *GENE ID* | *logFC* | *logCPM* | *LR* | *PValue* | *FDR* |
| ENSG00000168685.10 | IL7R | -3.58 | 5.69 | 25.92 | 3.56E-07 | 6.12E-04 |
| ENSG00000162631.13 | NTNG1 | -3.71 | 6.07 | 23.64 | 1.16E-06 | 1.63E-03 |
| ENSG00000141576.10 | RNF157 | -4.59 | 4.98 | 17.51 | 2.85E-05 | 1.92E-02 |
| ENSG00000100055.16 | CYTH4 | -5.02 | 3.61 | 18.48 | 1.72E-05 | 1.26E-02 |
| ENSG00000176204.9 | LRRTM4 | -5.07 | 3.99 | 16.63 | 4.54E-05 | 2.19E-02 |
| ENSG00000136960.7 | ENPP2 | -5.21 | 3.78 | 20.32 | 6.53E-06 | 5.94E-03 |
| ENSG00000107485.11 | GATA3 | -5.40 | 4.82 | 15.86 | 6.81E-05 | 2.64E-02 |
| ENSG00000132872.6 | SYT4 | -5.73 | 6.65 | 32.20 | 1.39E-08 | 2.15E-04 |
| ENSG00000115350.7 | POLE4 | -6.00 | 2.74 | 14.41 | 1.47E-04 | 4.89E-02 |
| ENSG00000116852.9 | KIF21B | -6.07 | 3.21 | 16.64 | 4.52E-05 | 2.19E-02 |
| ENSG00000154760.9 | SLFN13 | -6.37 | 3.39 | 17.57 | 2.77E-05 | 1.92E-02 |
| ENSG00000118507.11 | AKAP7 | -6.42 | 2.71 | 14.33 | 1.53E-04 | 4.89E-02 |
| ENSG00000173200.8 | PARP15 | -6.43 | 4.71 | 28.59 | 8.95E-08 | 4.61E-04 |
| ENSG00000048162.15 | NOP16 | -6.61 | 2.87 | 16.54 | 4.77E-05 | 2.23E-02 |
| ENSG00000143297.14 | FCRL5 | -6.65 | 4.36 | 15.93 | 6.56E-05 | 2.64E-02 |
| ENSG00000070190.8 | DAPP1 | -6.74 | 3.51 | 24.92 | 5.99E-07 | 9.25E-04 |
| ENSG00000238121.1 | LINC00426 | -6.75 | 3.34 | 14.31 | 1.55E-04 | 4.89E-02 |
| ENSG00000185404.12 | SP140L | -7.13 | 3.34 | 23.42 | 1.30E-06 | 1.67E-03 |
| ENSG00000161929.10 | C17orf87 | -7.14 | 2.79 | 15.00 | 1.08E-04 | 3.78E-02 |
| ENSG00000262714.1 | RP11-44F14.8 | -7.36 | 3.17 | 19.88 | 8.27E-06 | 6.72E-03 |
| ENSG00000234665.3 | RP11-262H14.*3* | -8.16 | 2.07 | 14.67 | 1.28E-04 | 4.40E-02 |
| ENSG00000239235.3 | AC008280.5 | -8.18 | 2.07 | 14.32 | 1.55E-04 | 4.89E-02 |
| ENSG00000156345.11 | CDK20 | -8.29 | 2.51 | 17.03 | 3.68E-05 | 2.19E-02 |
| ENSG00000232411.1 | AC009495.3 | -8.40 | 2.58 | 15.94 | 6.54E-05 | 2.64E-02 |
| ENSG00000240219.1 | RP11-430C7.5 | -8.40 | 2.12 | 15.74 | 7.25E-05 | 2.67E-02 |
| ENSG00000157315.4 | TMED6 | -8.44 | 2.06 | 15.81 | 7.02E-05 | 2.64E-02 |
| ENSG00000235532.1 | LINC00402 | -8.53 | 2.59 | 16.63 | 4.54E-05 | 2.19E-02 |
| ENSG00000054219.9 | LY75 | -8.53 | 2.90 | 21.98 | 2.75E-06 | 3.04E-03 |
| ENSG00000101311.11 | FERMT1 | -8.57 | 2.12 | 16.06 | 6.14E-05 | 2.64E-02 |
| ENSG00000246339.3 | EXTL3-AS1 | -8.60 | 2.64 | 19.70 | 9.05E-06 | 6.99E-03 |
| ENSG00000180938.5 | ZNF572 | -8.70 | 2.38 | 16.90 | 3.94E-05 | 2.19E-02 |
| ENSG00000203872.5 | C6orf163 | -8.76 | 2.27 | 16.15 | 5.84E-05 | 2.64E-02 |
| ENSG00000265625.1 | RP11-68I3.11 | -8.84 | 3.26 | 26.37 | 2.82E-07 | 5.69E-04 |
| ENSG00000153896.12 | ZNF599 | -8.87 | 3.07 | 21.81 | 3.01E-06 | 3.10E-03 |
| ENSG00000221817.3 | PPP3CB-AS1 | -8.95 | 3.12 | 22.27 | 2.37E-06 | 2.81E-03 |
| ENSG00000248329.1 | APELA | -9.09 | 2.97 | 20.86 | 4.94E-06 | 4.77E-03 |
| ENSG00000111358.8 | GTF2H3 | -9.22 | 3.17 | 26.28 | 2.95E-07 | 5.69E-04 |
| ENSG00000267002.1 | RP11.242D8.1 | -9.40 | 3.26 | 27.82 | 1.33E-07 | 5.13E-04 |
| ENSG00000204394.8 | VARS | -9.45 | 3.12 | 27.16 | 1.88E-07 | 5.69E-04 |
| ENSG00000261616.1 | RP11-6O2.3 | -9.79 | 2.79 | 16.85 | 4.05E-05 | 2.19E-02 |
| ENSG00000090402.3 | SI | -9.82 | 3.38 | 15.41 | 8.64E-05 | 3.11E-02 |
| ENSG00000184613.6 | NELL2 | -9.97 | 3.86 | 17.23 | 3.31E-05 | 2.13E-02 |
| ENSG00000267959.1 | MIR3188 | -10.15 | 3.28 | 15.82 | 6.96E-05 | 2.64E-02 |
| ENSG00000164122.4 | ASB5 | -10.56 | 3.87 | 19.98 | 7.83E-06 | 6.72E-03 |

logFC = log2 fold change between the groups, logCPM = the average log2-counts-per-million, LR = likelihood ratio statistics, *P* value = the two-sided *P* value, FDR = False discovery rate.

| **Table S12.** GSEA on Seeding versus Non-seeding foci | | | | | | |
| --- | --- | --- | --- | --- | --- | --- |
| Enriched in Seeding versus Non-seeding | | | | | | |
| **NAME** | **SIZE** | **ES** | **NES** | **NOM p-val** | **FDR q-val** | **FWER p-val** |
| HALLMARK_HEDGEHOG_SIGNALING | 20 | 0.51 | 1.60 | 2.72E-02 | 1.11E-01 | 1.41E-01 |
| HALLMARK_ESTROGEN_RESPONSE_EARLY | 136 | 0.34 | 1.63 | 2.14E-03 | 1.47E-01 | 9.70E-02 |
| HALLMARK_FATTY_ACID_METABOLISM | 113 | 0.33 | 1.51 | 7.84E-03 | 1.63E-01 | 2.80E-01 |
| HALLMARK_ANDROGEN_RESPONSE | 97 | 0.30 | 1.36 | 3.89E-02 | 2.26E-01 | 6.50E-01 |
| HALLMARK_UV_RESPONSE_UP | 108 | 0.30 | 1.35 | 3.38E-02 | 2.30E-01 | 7.06E-01 |
| Enriched in Seeding versus Non-seeding | | | | | | |
| **NAME** | **SIZE** | **ES** | **NES** | **NOM p-val** | **FDR q-val** | **FWER p-val** |
| HALLMARK_ALLOGRAFT_REJECTION | 133 | -0.42 | -1.94 | 0.00E+00 | 2.38E-03 | 4.00E-03 |
| HALLMARK_KRAS_SIGNALING_UP | 122 | -0.34 | -1.54 | 3.87E-03 | 7.28E-02 | 2.42E-01 |
| Size = number of genes in gene set, ES = enrichment score, NES = normalized ES, NOM *P* value = the statistical significance of the enrichment score, FDR q-value = the estimated probability that the normalized enrichment score represents a false positive finding, FDR = False discovery rate, FWER *P* value = familywise-error rate, a more conservatively estimated probability that the NES represents a false positive finding. | | | | | | |
|  |  |  |  |  |  |  |

**References**

1. Moller, M., et al., *Heterogeneous patterns of DNA methylation-based field effects in histologically normal prostate tissue from cancer patients.* Sci Rep, 2017. **7**: p. 40636.

2. Kim, D., et al., *TopHat2: accurate alignment of transcriptomes in the presence of insertions, deletions and gene fusions.* Genome Biol, 2013. **14**(4): p. R36.

3. Langmead, B. and S.L. Salzberg, *Fast gapped-read alignment with Bowtie 2.* Nat Methods, 2012. **9**(4): p. 357-9.

4. *Broad Institute. Picard tools at* [*http://broadinstitute.github.io/picard*](http://broadinstitute.github.io/picard)*.*

5. Trapnell, C., et al., *Transcript assembly and quantification by RNA-Seq reveals unannotated transcripts and isoform switching during cell differentiation.* Nat Biotechnol, 2010. **28**(5): p. 511-5.

6. Anders, S., P.T. Pyl, and W. Huber, *HTSeq--a Python framework to work with high-throughput sequencing data.* Bioinformatics, 2015. **31**(2): p. 166-9.

7. Team, R.D.C., *R: A language and environment for statistical computing. .* R Foundation for Statistical Computing, 2008. **ISBN 3-900051-07-0**.

8. Robinson, M.D., D.J. McCarthy, and G.K. Smyth, *edgeR: a Bioconductor package for differential expression analysis of digital gene expression data.* Bioinformatics, 2010. **26**(1): p. 139-40.

9. Merola, R., et al., *PCA3 in prostate cancer and tumor aggressiveness detection on 407 high-risk patients: a National Cancer Institute experience.* J Exp Clin Cancer Res, 2015. **34**: p. 15.

10. Vaarala, M.H., et al., *Identification of androgen-regulated genes in human prostate.* Mol Med Rep, 2012. **6**(3): p. 466-72.

11. Li, W., et al., *Diagnostic significance of overexpression of Golgi membrane protein 1 in prostate cancer.* Urology, 2012. **80**(4): p. 952 e1-7.

12. Varambally, S., et al., *Golgi protein GOLM1 is a tissue and urine biomarker of prostate cancer.* Neoplasia, 2008. **10**(11): p. 1285-94.

13. Luo, J., et al., *Alpha-methylacyl-CoA racemase: a new molecular marker for prostate cancer.* Cancer Res, 2002. **62**(8): p. 2220-6.

14. Magi-Galluzzi, C., et al., *Alpha-methylacyl-CoA racemase: a variably sensitive immunohistochemical marker for the diagnosis of small prostate cancer foci on needle biopsy.* Am J Surg Pathol, 2003. **27**(8): p. 1128-33.

15. Xiao, L., et al., *The Germ Cell Gene TDRD1 as an ERG Target Gene and a Novel Prostate Cancer Biomarker.* Prostate, 2016. **76**(14): p. 1271-84.

16. Wang, N., et al., *Autocrine Activation of CHRM3 Promotes Prostate Cancer Growth and Castration Resistance via CaM/CaMKK-Mediated Phosphorylation of Akt.* Clin Cancer Res, 2015. **21**(20): p. 4676-85.

17. Zhao, J., et al., *Alterations of androgen receptor-regulated enhancer RNAs (eRNAs) contribute to enzalutamide resistance in castration-resistant prostate cancer.* Oncotarget, 2016. **7**(25): p. 38551-38565.

18. Grasso, C.S., et al., *The mutational landscape of lethal castration-resistant prostate cancer.* Nature, 2012. **487**(7406): p. 239-43.

19. Adamo, P. and M.R. Ladomery, *The oncogene ERG: a key factor in prostate cancer.* Oncogene, 2016. **35**(4): p. 403-14.

20. Hagglof, C., et al., *TMPRSS2-ERG expression predicts prostate cancer survival and associates with stromal biomarkers.* PLoS One, 2014. **9**(2): p. e86824.

21. Chiang, Y.T., et al., *Prostate cancer metastasis-driving genes: hurdles and potential approaches in their identification.* Asian J Androl, 2014. **16**(4): p. 545-8.

22. Alinezhad, S., et al., *Validation of Novel Biomarkers for Prostate Cancer Progression by the Combination of Bioinformatics, Clinical and Functional Studies.* PLoS One, 2016. **11**(5): p. e0155901.

23. Ribeiro, F.R., et al., *Cysteine-rich secretory protein-3 (CRISP3) is strongly up-regulated in prostate carcinomas with the TMPRSS2-ERG fusion gene.* PLoS One, 2011. **6**(7): p. e22317.

24. Al Bashir, S., et al., *Cysteine- rich secretory protein 3 (CRISP3), ERG and PTEN define a molecular subtype of prostate cancer with implication to patients' prognosis.* J Hematol Oncol, 2014. **7**: p. 21.

25. Signoretti, S., et al., *p63 is a prostate basal cell marker and is required for prostate development.* Am J Pathol, 2000. **157**(6): p. 1769-75.

26. Parsons, J.K., et al., *p63 protein expression is rare in prostate adenocarcinoma: implications for cancer diagnosis and carcinogenesis.* Urology, 2001. **58**(4): p. 619-24.

27. Tan, H.L., et al., *Prostate adenocarcinomas aberrantly expressing p63 are molecularly distinct from usual-type prostatic adenocarcinomas.* Mod Pathol, 2015. **28**(3): p. 446-56.

28. Chandran, U.R., et al., *Gene expression profiles of prostate cancer reveal involvement of multiple molecular pathways in the metastatic process.* BMC Cancer, 2007. **7**: p. 64.

29. Mitra, R. and O.B. Goodman, Jr., *CYP3A5 regulates prostate cancer cell growth by facilitating nuclear translocation of AR.* Prostate, 2015. **75**(5): p. 527-38.

30. Verdoodt, B., et al., *MicroRNA-205, a novel regulator of the anti-apoptotic protein Bcl2, is downregulated in prostate cancer.* Int J Oncol, 2013. **43**(1): p. 307-14.

31. Kanno, Y., et al., *TRIM29 as a novel prostate basal cell marker for diagnosis of prostate cancer.* Acta Histochem, 2014. **116**(5): p. 708-12.

32. Kulkarni, P., et al., *Prostate-associated gene 4 (PAGE4), an intrinsically disordered cancer/testis antigen, is a novel therapeutic target for prostate cancer.* Asian J Androl, 2016. **18**(5): p. 695-703.

33. Varisli, L., *Identification of new genes downregulated in prostate cancer and investigation of their effects on prognosis.* Genet Test Mol Biomarkers, 2013. **17**(7): p. 562-6.

34. Strand, S.H., et al., *RHCG and TCAF1 promoter hypermethylation predicts biochemical recurrence in prostate cancer patients treated by radical prostatectomy.* Oncotarget, 2017. **8**(4): p. 5774-5788.

35. Schleicher, R.L., et al., *Neurofilament heavy chain-like messenger RNA and protein are present in benign prostate and down-regulated in prostatic carcinoma.* Cancer Res, 1997. **57**(16): p. 3532-6.

36. Xu, A. and S. Sun, *Genomic profiling screens small molecules of metastatic prostate carcinoma.* Oncol Lett, 2015. **10**(3): p. 1402-1408.

37. Love, H.D., et al., *Androgen regulated genes in human prostate xenografts in mice: relation to BPH and prostate cancer.* PLoS One, 2009. **4**(12): p. e8384.

38. Sertkaya, S., et al., *Decreased expression of EFS is correlated with the advanced prostate cancer.* Tumour Biol, 2015. **36**(2): p. 799-805.

39. Sandsmark, E., et al., *A novel non-canonical Wnt signature for prostate cancer aggressiveness.* Oncotarget, 2017. **8**(6): p. 9572-9586.

40. Siu, M.K., et al., *TCF7 is suppressed by the androgen receptor via microRNA-1-mediated downregulation and is involved in the development of resistance to androgen deprivation in prostate cancer.* Prostate Cancer Prostatic Dis, 2017. **20**(2): p. 172-178.

41. Lapointe, J., et al., *Gene expression profiling identifies clinically relevant subtypes of prostate cancer.* Proc Natl Acad Sci U S A, 2004. **101**(3): p. 811-6.

42. Franzen, C.A., et al., *Matrix protein CCN1 is critical for prostate carcinoma cell proliferation and TRAIL-induced apoptosis.* Mol Cancer Res, 2009. **7**(7): p. 1045-55.

43. Ramaswamy, S., et al., *A molecular signature of metastasis in primary solid tumors.* Nat Genet, 2003. **33**(1): p. 49-54.

44. Luca, B.A., et al., *DESNT: A Poor Prognosis Category of Human Prostate Cancer.* Eur Urol Focus, 2017.

45. Luo, G., et al., *Long Non-Coding RNA MEG3 Inhibits Cell Proliferation and Induces Apoptosis in Prostate Cancer.* Cell Physiol Biochem, 2015. **37**(6): p. 2209-20.

46. Hsing, A.W., et al., *Polymorphic markers in the SRD5A2 gene and prostate cancer risk: a population-based case-control study.* Cancer Epidemiol Biomarkers Prev, 2001. **10**(10): p. 1077-82.

47. Pascal, L.E., et al., *Gene expression down-regulation in CD90+ prostate tumor-associated stromal cells involves potential organ-specific genes.* BMC Cancer, 2009. **9**: p. 317.

48. Wang, Z., et al., *Loss of ATF3 promotes Akt activation and prostate cancer development in a Pten knockout mouse model.* Oncogene, 2015. **34**(38): p. 4975-84.

49. Wang, Z. and C. Yan, *Emerging roles of ATF3 in the suppression of prostate cancer.* Mol Cell Oncol, 2016. **3**(1): p. e1010948.

50. Vanaja, D.K., et al., *PDLIM4 repression by hypermethylation as a potential biomarker for prostate cancer.* Clin Cancer Res, 2006. **12**(4): p. 1128-36.

51. Yu, L., et al., *Repression of NR4A1 by a chromatin modifier promotes docetaxel resistance in PC-3 human prostate cancer cells.* FEBS Lett, 2013. **587**(16): p. 2542-51.

52. Liu, P., et al., *Sex-determining region Y box 4 is a transforming oncogene in human prostate cancer cells.* Cancer Res, 2006. **66**(8): p. 4011-9.

53. Turashvili, G., et al., *Novel markers for differentiation of lobular and ductal invasive breast carcinomas by laser microdissection and microarray analysis.* BMC Cancer, 2007. **7**: p. 55.

54. Acevedo, V.D., et al., *Inducible FGFR-1 activation leads to irreversible prostate adenocarcinoma and an epithelial-to-mesenchymal transition.* Cancer Cell, 2007. **12**(6): p. 559-71.

55. Smid, M., et al., *Subtypes of breast cancer show preferential site of relapse.* Cancer Res, 2008. **68**(9): p. 3108-14.

56. Jaeger, J., et al., *Gene expression signatures for tumor progression, tumor subtype, and tumor thickness in laser-microdissected melanoma tissues.* Clin Cancer Res, 2007. **13**(3): p. 806-15.
